# The multiple functions of miR-574-5p in the neuroblastoma tumor microenvironment

**DOI:** 10.3389/fphar.2023.1183720

**Published:** 2023-09-04

**Authors:** Eva Proestler, Julia Donzelli, Sheila Nevermann, Kai Breitwieser, Leon F. Koch, Tatjana Best, Maria Fauth, Malin Wickström, Patrick N. Harter, Per Kogner, Grégory Lavieu, Karin Larsson, Meike J. Saul

**Affiliations:** ^1^ Fachbereich Biologie, Technische Universität Darmstadt, Darmstadt, Germany; ^2^ Merck KGaA, Darmstadt, Germany; ^3^ Prolytic GmbH, a Kymos Company, Frankfurt, Germany; ^4^ Childhood Cancer Research Unit, Department of Children’s and Women’s Health, Karolinska Institutet, Stockholm, Sweden; ^5^ Institute of Neurology (Edinger-Institute), University Hospital Frankfurt, Goethe University, Frankfurt am Main, Frankfurt, Germany; ^6^ German Cancer Consortium (DKTK), Heidelberg, Germany; ^7^ German Cancer Research Center (DKFZ), Heidelberg, Germany; ^8^ Frankfurt Cancer Institute (FCI), Frankfurt am Main, Frankfurt, Germany; ^9^ INSERM U1316, UMR7057, Centre National de la Recherche Scientifique (CNRS), Université Paris Cité, Paris, France; ^10^ Rheumatology Unit, Department of Medicine, Karolinska University Hospital, Stockholm, Sweden

**Keywords:** miR-574-5p, TLR7/8, PGE2, tetraspanins, neuroblastoma, NSCLC

## Abstract

Neuroblastoma is the most common extracranial solid tumor in childhood and arises from neural crest cells of the developing sympathetic nervous system. Prostaglandin E_2_ (PGE_2_) has been identified as a key pro-inflammatory mediator of the tumor microenvironment (TME) that promotes neuroblastoma progression. We report that the interaction between the microRNA miR-574-5p and CUG-binding protein 1 (CUGBP1) induces the expression of microsomal prostaglandin E_2_ synthase 1 (mPGES-1) in neuroblastoma cells, which contributes to PGE_2_ biosynthesis. PGE_2_ in turn specifically induces the sorting of miR-574-5p into small extracellular vesicles (sEV) in neuroblastoma cell lines. sEV are one of the major players in intercellular communication in the TME. We found that sEV-derived miR-574-5p has a paracrine function in neuroblastoma. It acts as a direct Toll-like receptor 7/8 (TLR7/8) ligand and induces α-smooth muscle actin (α-SMA) expression in fibroblasts, contributing to fibroblast differentiation. This is particularly noteworthy as it has an opposite function to that in the TME of lung carcinoma, another PGE_2_ dependent tumor type. Here, sEV-derived miR-574-5p has an autokrine function that inhibits PGE_2_ biosynthesis in lung cancer cells. We report that the tetraspanin composition on the surface of sEV is associated with the function of sEV-derived miR-574-5p. This suggests that the vesicles do not only transport miRs, but also appear to influence their mode of action.

## 1 Introduction

Neuroblastoma is the most common extracranial solid tumor in children under 1 year of age and arises from neural crest cells of the developing sympathetic nervous system (Matthay et al., 2016). It has a remarkable phenotypic heterogeneity, ranging from spontaneous regression in the absence of treatment to a relentlessly progressive disease that is resistant to intensive multimodal therapy (Baker et al., 2010; Nuchtern et al., 2012; Pinto et al., 2015). Despite significant advances in cancer treatment, survival rates for high-risk neuroblastoma patients remain low (Smith and Foster, 2018). Standard treatments for neuroblastoma, such as chemotherapy and radiotherapy, target the proliferating, genetically unstable tumor cells. However, resistance to therapy is inevitable after long-term treatment, leading to treatment failure and cancer relapse (Larsson et al., 2019; Zhou et al., 2023). Therefore, a better understanding of how neuroblastoma cells communicate with the tumor microenvironment (TME) is essential for the development of more effective targeted therapies. The TME is generally composed of different cell types, including fibroblasts, immune cells and structural elements. Intratumoral interactions between cancer cells and TME cells contribute to cancer progression at different stages (Belli et al., 2018; Neophytou et al., 2021). Communication between cancer cells and TME cells occurs mainly through contact-independent mechanisms and is mediated by soluble proteins such as growth factors, cytokines and chemokines. Recent evidence suggests that other soluble factors such as extracellular vesicles may be involved and can contribute to neuroblastoma progression (Blavier et al., 2020; Marimpietri et al., 2021). Small extracellular vesicles (sEV) of endosomal origin are actively secreted by all cell types (Raposo and Stoorvogel, 2013; Witwer and Théry, 2019) and are critical regulators of intercellular communication in these processes (Penfornis et al., 2016; Mathieu et al., 2019; Mir and Goettsch, 2020). Neuroblastoma cells release sEV as a mechanism for the abrogation of the antitumor immune response (Marimpietri et al., 2021). These sEV promote a tolerogenic microenvironment in both primary tumors and metastases (Morandi et al., 2019; Marimpietri et al., 2021). In addition, neuroblastoma cell-derived sEV are involved in modulating the antitumor T-cell response, and such immunosuppressive activity must be considered when developing immunotherapeutic strategies (Ali et al., 2020). SEV contain various bioactive molecules that can be taken up by neighboring or distant cells far from their release. This modulates the behavior of the recipient cells to their bioactive compounds (Zhang et al., 2019).

One of these components are microRNAs (miRNAs, miRs), a group of small non-coding RNAs that have multiple effects on gene regulation (Bartel, 2004; Cheng et al., 2014). Many of these miRNAs are involved in a complex regulatory network for the modulation of various biological functions, including inflammatory processes through the modulation of lipid mediators (Saul et al., 2019a). In general, the sEV envelope protects miRNAs from degradation (Cheng et al., 2014; Hegewald et al., 2020) and can influence the internalization by recipient cells (Rana et al., 2012). By modulating the physiological function of their target cell in the tumor microenvironment or in distant organs, it critically affects tumor progression (Dai et al., 2020). In addition, chronic inflammation is known to promote a microenvironment that triggers tumor development (Mantovani et al., 2008; Greten and Grivennikov, 2019). The mechanisms by which inflammatory factors influence the TME and promote tumor growth are not fully understood. However, there is increasing evidence that the lipid mediator prostaglandin (PG) E_2_ plays a central role in these processes (Samuelsson et al., 2007; Nakanishi and Rosenberg, 2013). In general, PGE_2_ is formed from arachidonic acid in a two-step enzymatic reaction in which arachidonic acid is first converted to PGH_2_ by cyclooxygenases and then to PGE_2_ by microsomal prostaglandin E synthase-1 (mPGES-1) (Murakami et al., 2000; Wang and Dubois, 2006). PGE_2_ binds to specific prostaglandin E_2_ receptors EP1 to 4, which belong to the G-protein coupled receptor family. Each receptor mediates the tumor-promoting properties of PGE_2_ through distinct intracellular signaling pathways (Pai et al., 2002). Cancer-associated fibroblasts are the main source of PGE_2_ in neuroblastoma, particularly in a genetic subtype of neuroblastoma with 11q deletion, promoting angiogenesis, immunosuppression and tumor growth (Larsson et al., 2015; Larsson et al., 2019).Targeting this inflammatory pathway offers a therapeutic option for neuroblastoma (Larsson et al., 2015; Larsson et al., 2019) and other cancers (Harris et al., 2005; Cai et al., 2020; Drew et al., 2020). Blocking PGE_2_ production not only sensitizes cancer cells to chemotherapeutics (Hanaka et al., 2009), but also reduces programmed cell death ligand 1 expression, alleviates immunosuppression, and stimulates anti-tumor immune responses (Prima et al., 2017). Therefore, combining standard cancer therapies with PGE_2_ inhibition is a promising anti-cancer strategy (Zelenay et al., 2015).

Little is known to date about the interaction of these signaling pathways, given the importance of PGE_2_ and sEV in the TME. Considering this, the recent discovery that PGE_2_ stimulates the sorting of the miRNA miR-574-5p into sEV in non-small cell lung cancer (NSCLC) is noteworthy (Donzelli et al., 2021). PGE_2_ is thus the first known exogenous factor to modulate specifically miRNA secretion in sEV, which is mediated by EP1 and 3 (Donzelli et al., 2021). After internalization by target cells, sEV-derived miR-574-5p activates Toll-like receptors (TLR) 7/8 and decreases PGE_2_-biosynthesis in adenocarcinoma cells, but not in squamous-cell carcinoma cells. This function stands in contrast to intracellular miR-574-5p. MiR-574-5p induces the expression of microsomal prostaglandin E_2_ synthase-1 (mPGES-1) by interacting with the RNA binding protein CUG binding protein 1 (CUGBP1) (Saul et al., 2019b; Emmerich et al., 2020). Thus, miR-574-5p acts as decoy for CUGBP1. It prevents CUGBP1 from binding to the mPGES-1 3′untranslated region (UTR), resulting in the generation of a mPGES-1 3′UTR isoform with higher translational efficiency derived from alternative splicing of mPGES-1 wild type (WT) mRNA (Saul et al., 2019b).

The extent to which this regulatory mechanism can be generalized to other PGE_2_-dependent tumors is currently unknown. Neuroblastoma is a unique model to study the role of miR-574-5p in PGE_2_-driven cancer progression in non-epithelial tumors and an important complementary model to NSCLC. Therefore, we decided to further investigate the role of miR-574-5p in the neuroblastoma microenvironment in terms of its intracellular function as a regulator of PGE_2_ biosynthesis and its extracellular function as a TLR7/8 ligand. In addition, we aimed to determine whether the function of sEV-derived miR-574-5p in TME is comparable to that in NSCLC or whether miR-574-5p affects TME differently in neuroblastoma. In addition, we examined whether certain properties of the sEV envelope might influence the function of sEV-derived miR-574-5p as a TLR7/8 ligand.

## 2 Materials and methods

### 2.1 Neuroblastoma tumor tissue

Neuroblastoma tumor tissue samples were provided by Prof. Dr. Per Kogner, Karolinska Institutet, Stockholm, Sweden. Ethical approval was obtained by the Stockholm Regional Ethical Review Board and the Karolinska University Hospital Research Ethics Committee (approval nos. 2009/1369-31/1 and 03/736). Tissue samples of in total 20 patients from different neuroblastoma subtypes were stained: *MYCN* amplified (N = 8), 11q deleted (N = 5), low risk (N = 5) and 11q deleted/*MYCN* amplified (N = 2).

### 2.2 Immunohistochemistry staining of tissue sections

Immunohistochemistry (IHC) staining of mPGES-1 and CUGBP1 was performed as previously described (Donzelli et al., 2021). For this purpose, rabbit-α-mPGES-1 (Cayman Chemicals, Ann Arbor, United States, cay160140, 1:200) and rabbit-α-CUGBP1 (Abcam, Cambridge, United Kingdom, ab129115, 1:100) antibodies were used and developed using a 3,3′-Diaminobenzidine substrate kit (Abcam, Cambridge, United Kingdom) following the manufacturer’s instructions. Counterstaining was performed with hematoxylin (Sigma-Aldrich, St. Louis, United States) for 15 s.

### 2.3 *In situ* hybridization of tissue sections

*In situ* hybridization (ISH) was performed as previously described (Donzelli et al., 2021). Specific locked nucleic acid probes (Qiagen, Hilden, GER) which were double-labeled with fluorescein against miR-574-5p were used. Control *in situ* hybridization was performed using a digoxygenin-labeled scramble control probe.

### 2.4 Cell lines and cell culture conditions

According to the MISEV2018 guidelines (Thery et al., 2018), all cell culture experiments were carried out with sEV-depleted fetal calf serum (FCS, Sigma-Aldrich, St. Louis, United States) which was centrifuged at 120 000 x g, 4°C for 18 h in an Optima™ XPN-80 ultracentrifuge (Beckman Coulter, Brea, United States). Subsequently, the bottom third of the volume was discarded to exclude contamination by endogenous sEV. All cell culture experiments were carried out under sterile and standardized cell culture conditions (37°C, 5% CO_2_ and 98% humidity). The two neuroblastoma cell lines SK-N-AS (ATCC:CRL-2137™) and SK-N-SH (RRID:CVCL_0531) and the human lung adenocarcinoma cell line A549 (ATCC: CCL-185™) were cultured in Dulbecco’s modified Eagle medium (DMEM, Thermo Fisher Scientific, Waltham, United States) with 10% (v/v) heat-inactivated FCS, 100 U/mL Penicillin (gibco, Thermo Fisher Scientific, Waltham, United States), 100 μg/mL Streptomycin (gibco, Thermo Fisher Scientific, Waltham, United States) and 1 mM sodium pyruvate (Thermo Fisher Scientific, Waltham, United States). The human fetal lung fibroblast cell line HFL1 (CCL153, ATCC, Manassas, United States) was cultured in Kaighn’s modification of Ham’s F-12 medium (F-12K, gibco, Thermo Fisher Scientific, Waltham, United States) with 10% (v/v) FCS, 100 U/mL Penicillin and 100 μg/mL Streptomycin. The human pulmonary squamous cell carcinoma cell line 2106T (CLS Cell Lines Service GmbH, Eppelheim, GER) was cultured in 50% DMEM and 50% F-12K with 5% (v/v) FCS, 100 U/mL Penicillin, and 100 μg/mL Streptomycin, 0.5 mM sodium pyruvate and 15 mM 4-(2-hydroxyethyl)-1-piperazineethanesulfonic acid (HEPES, Sigma-Aldrich, St. Louis, United States). All cell lines were passaged twice weekly. The HFL1 cell line was used for experiments from passages 4 to 11. For sEV-derived miR-574-5p measurements, cells were stimulated with 5 nM PGE_2_, 5 nM Butaprost, 5 nM Sulprostone (all Sigma-Aldrich, St. Louis, United States), 5 nM L-902,688 (Cayman Chemicals, Ann Arbor, United States), or vehicle dimethyl sulfoxide (DMSO, Carl Roth, Karlsruhe, GER) for 30 min, 1 h, and 2 h. HFL1 were stimulated with 2 μg/mL purified sEV, 100 ng/mL Resiquimod (R848, Invivogen, San Diego, United States), or 200 nM ODN 2088 Control (ODN 2087) (Miltenyi Biotec, Bergisch-Gladbach, GER) for 72 h.

### 2.5 3D cell culture

Spheroid cultures of A549, SK-N-AS, and HFL1 cells were generated in hanging drops of DMEM, supplemented with 0.4% methylcellulose (Foty, 2011) (Sigma-Aldrich, St. Louis, United States). Each hanging drop contained a total cell number of 1 × 10^4^ cells in a volume of 50 μL medium. For co-culture experiments, 66% SK-N-AS cells were combined with 33% HFL1 cells. Spheroids were cultured for 72 h and then stimulated with 5 ng/mL interleukin (IL)-1β for 24 h.

### 2.6 RNA extraction

Total RNA from cells was extracted using TRIzol™ reagent (Thermo Fisher Scientific, Waltham, United States) and digested with Turbo™ DNase (Thermo Fisher Scientific, Waltham, United States) according to the manufacturer’s instructions. 1 μg of DNase-treated RNA was used for reverse transcription using the High-Capacity RNA-to-cDNA Kit (Thermo Fisher Scientific, Waltham, United States) according to the manufacturer’s instructions. RNA from sEV was extracted using a phenol/guanidinium thiocyanate (GTC)-based extraction method. To each purified sEV sample, 200 μL extraction buffer (pH 4.8, 150 nM sucrose, 10 mM sodium acetate), 20 μL sodium dodecyl sulfate (SDS, 20%, Carl Roth, Karlsruhe, GER), 200 μL 6 M GTC (Sigma-Aldrich, St. Louis, United States) and 200 μL Roti®-Aqua- phenol (Carl Roth, Karlsruhe, GER), pre-warmed to 65°C, were added to each sample. The samples were vortexed and incubated at 65°C for 5 min. Afterward, 2 nmol synthetic ath-miR-159 and 200 nmol of cel-miR-39-3p were spiked in as internal standards for normalization and to enhance precipitation efficiency (Fauth et al., 2019). 200 μL chloroform isoamylalcohol (1:24, both Carl Roth, Karlsruhe, GER) were mixed into each tube and samples were centrifuged 5 min at 13 000 x g. The upper aqueous phase was transferred to fresh tubes and 1.5 mL ethanol (EtOH, 100%), 50 μL 3 M sodium acetate and 1 μL GlycoBlue™ were added. RNA was precipitated at −80°C for 20 min and tubes were centrifuged for 20 min. The supernatant was discarded, pellets were resuspended in 17 μL MQ and all samples were digested with Turbo™ DNase. The reaction was stopped by adding 100 μL EtOH (100%), 2 μL 3 M sodium acetate and 1 μL GlycoBlue™. After precipitation at −80°C for 20 min, samples were centrifuged 20 min, the supernatant was discarded and pellets washed with 200 μL EtOH (70%). After a final centrifugation step for 10 min, the supernatant was discarded, and pellets resuspended in 15 μL MQ. 10 μL of each sample were reverse transcribed using miRCURY LNA RT (Qiagen, Hilden, GER) following manufacturer’s instructions and measured by reverse transcription quantitative polymerase chain reaction (RT-qPCR).

### 2.7 RT-qPCR

MiRs from RNA immunoprecipitation, 3D cell culture experiments and RNase and Triton X-100 treatment of sEV were analyzed with the miRCURY system (Qiagen, Hilden, GER) and the primers miR-574-5p (YCP0044301) and ath-miR-159a (YCP0044303) following the manufacturer’s instructions. For all other samples 2 µL of each RNA sample were incubated for elongation with 2 U *E.coli* Poly(A) Polymerase (New England Biolabs GmbH, Ipswich, United States) and transcription by 100 U M-MuLV Reverse Transcriptase (New England Biolabs GmbH, Ipswich, United States) for 1 h at 42°C. RT-qPCR analysis was performed using the PerfeCTa^®^ SYBR^®^ Green SuperMix (Quantabio, Beverly, United States) according to the manufacturer`s instructions. MRNA transcripts were analyzed as previously described (Donzelli et al., 2021) with the following primer pairs: mPGES-1 coding sequence (mPGES-1 CDS fwd: 5′-GAA​GAA​GGC​CTT​TGC​CAA​C-3′; mPGES-1 CDS rev: 5′-CCA​GGA​AAA​GGA​AGG​GGT​AG-3′), mPGES-1 (mPGES-1 fwd: 5′-TCC​CGG​GCT​AAG​AAT​GCA-3′; mPGES-1 rev: 5′-ATT​GGC​TGG​GCC​AGA​ATT​TC-3′), mPGES-1 3′UTR isoform (mPGES-1 iso fwd: 5′-GTG​CCC​GTG​TGT​GTG​TAT​GTG​TGT​GTG​TGT-3′; mPGES-1 iso rev: 5′-CCC​AGC​TGG​CAG​ACA​CTT​CCA​TTT​AAT​GAC​T-3′), CUGBP1 (CUGBP1 fwd: 5′-AAA​GTC​CTC​CCA​GGG​ATG​CA-3′; CUGBP1 rev: 5′-AGC​TTC​CTG​TCT​TCC​ACT​GCA​T-3′), COX-2 (COX-2 fwd: 5′-CCG​GGT​ACA​ATC​GCA​CTT​AT-3′; COX-2 rev: 5′-GGC​GCT​CAG​CCA​TAC​AG-3′), nervous system overexpressed protein 20 (NOXP20) (NOXP20 fwd: 5′-GGC​AAA​TCT​CTG​CTG​TCG​TC-3′; NOXP20 rev: 5′-CCT​GCT​TTT​TCC​TTG​ACT​GC-3′), α-Smooth Muscle Actin (α-SMA) (α-SMA fwd: 5′-GCT​GTT​TTC​CCA​TCC​ATT​GT-3’; α-SMA rev 5′-TTT​GCT​CTG​TGC​TTC​GTC​AC-3′). In all RT-qPCR measurements, GAPDH (GAPDH fwd: 5′-TGA​GAA​CGG​GAA​GCT​TGT​CA-3′; GAPDH rev: 5′-ATC​GCC​CCA​CTT​GAT​TTT​GG-3′) was used as an endogenous control to normalize cDNA quantities between different samples.

### 2.8 Western blotting

Western blot analysis was performed as previously described (Saul et al., 2019b). In brief, cells or spheroids were harvested, lysed with a tissue protein extraction reagent (T-PER, Thermo Fisher Scientific, Waltham, United States) and protein concentrations were determined via Bradford assay (Bio-Rad Laboratories, Hercules, United States). Then, 20–40 µg of total protein were separated on 12% SDS gels and afterwards, wet blotted on a nitrocellulose membrane (Sigma-Aldrich, St. Louis, United States). Membranes were incubated with antibodies against mPGES-1 (1:200, 160140, Cayman Chemicals, Ann Arbor, United States), CUGBP1 (1:500, ab129115, Abcam, Cambridge, United Kingdom), α-SMA (1:1000, ab7817, Abcam, Cambridge, United Kingdom) and GAPDH (1:1000, 2118, Cell Signaling Technology, Danvers, United States) as an internal standard at 4°C overnight, then incubated with suitable infrared dye conjugated secondary antibodies (IRDye^®^, LI-COR Biosciences, Lincoln, United States) for 45 min at RT. Protein bands were detected with the Odyssey Fc chemiluminescence reader (LI-COR Biosciences, Lincoln, United States), which provided consistent and reliable data for quantification even at low protein levels. For tetraspanin analysis in sEV, purified sEV from 30 mL culture medium and corresponding cells were lysed in RIPA buffer (50 mM Tris-HCl (Carl Roth, Karlsruhe, GER); 150 mM NaCl (MilliporeSigma, Burlington, United States); 1% Triton X-100 (Carl Roth, Karlsruhe, GER), 0.1% SDS, 0.1% deoxycholate acid (MilliporeSigma, Burlington, United States)) supplemented with EDTA-free protease inhibitor (cOmplete Mini, EDTA-free, Roche, Basel, Switzerland). Protein concentrations of cell lysate were determined via BCA assay (EMD Millipore Corp., Burlington, United States) and 50 µg of protein were loaded. Separation, blotting and staining were performed as described above. For the detection of tetraspanins, SDS-PAGE was performed under unreduced conditions and antibodies against CD9 (1:500, Clone ALB6, sc-59140, Santa Cruz Biotechnology, Dallas, United States), CD63 (1:1000, Clone H5C6, NBP2-42225, Novus Biologicals, Littleton, United States) and CD81 (1:1000, Clone 5A6, MABF 2061, Merck, Darmstadt, Germany) were used. Calnexin (1:2000, C4731, Sigma-Aldrich, St. Louis, United States), Syntenin-1 (1:500, sc-515538, Santa Cruz Biotechnology, Dallas, United States) and GAPDH were detected under reduced conditions. Odyssey NEWBLOT IR Stripping buffer (LI-COR Biosciences, Lincoln, United States) was used for membrane stripping according to manufacturer’s instructions.

### 2.9 RNA immunoprecipitation

For each condition, 6 × 10^6^ SK-N-AS cells were seeded in a 15 cm petri dish for 24 h and then stimulated with 5 ng/mL IL-1β for 24 h. Then, cells were washed with ice-cold 1x PBS (gibco, Thermo Fisher Scientific, Waltham, United States) and harvested by scraping with a cell scraper in 1x PBS with an EDTA-free protease inhibitor (Roche, Basel, CHE). Cells were pelleted by centrifugation for 5 min at 400 x g and 4°C and then resuspended in 1 mL lysis buffer containing 10 mM Tris-HCl, pH 7.5, 10 mM KCl (MilliporeSigma, Burlington, United States), 1.5 mM MgCl_2_, 0.5 mM DTT (MilliporeSigma, Burlington, United States), 0.9% IGEPAL CA-630 NP-40 (Merck, Darmstadt, GER), 8000 U RNase inhibitor, and EDTA-free protease inhibitor. The samples were incubated on ice for 10 min, and the cells were disrupted by ultrasonication 3 times for 10 s at 30% amplitude, with a 30 s pause in between. Next, samples were centrifuged at 10 000 x g for 10 min at 4°C. Finally, the supernatant containing total protein was transferred to a new tube, and 10% was taken as an input sample.

GammaBind Plus Sepharose beads (GE Healthcare, Freiburg, GER) were blocked for 90 min at 4°C in blocking buffer (0.2 mg/mL bovine serum albumin (BSA), 0.1 mg/mL yeast tRNA in 1x PBS). Then, the beads were washed 3 times with lysis buffer and centrifuged at 300 *g* for 5 min in-between. 10 μg antibodies against CUGBP1 (05-621 clone3B1; Merck, Darmstadt, GER) or normal mouse IgG antibody (12-371; Merck, Darmstadt, GER) were added to 50 µl beads and incubated rotating for 60 min at 4°C. The lysate was devided equally to the CUGBP1-/IgG-bead mixture and the samples were incubated at 4°C for 2 h for immunoprecipitation. Afterward, samples were washed with each wash buffer B1 (20 mM Tris-HCl pH 7.5, 150 mM NaCl, 2 mM EDTA (MilliporeSigma, Burlington, United States), 0.1% SDS, 1% Triton, and EDTA-free protease inhibitor), B2 (20 mM Tris-HCl pH 7.5, 500 mM NaCl, 2 mM EDTA, 0.1% SDS, 1% Triton and EDTA-free protease inhibitor) and B3 (10 mM Tris-HCl pH 7.5, 250 mM LiCl (MilliporeSigma, Burlington, United States), 1 mM EDTA, 1% sodium deoxycholate, 0.9% IGEPAL CA-630 NP-40, and EDTA-free protease inhibitor) for 5 min at 4°C, with centrifugation steps of 5 min and 300 x g in between. After the last washing step, 10% of each precipitate were taken for Western blot analysis to validate the immunoprecipitation. Western blot analysis was performed as previously described and CUGBP1 (1:1000, Abcam, Cambridge, United Kingdom, ab129115) antibody was used. The remaining precipitates were resuspended in TRIzol™ reagent (Thermo Fisher Scientific, Waltham, United States), and RNA was isolated as described above.

### 2.10 sEV purification

Cell culture supernatants were centrifuged at 2,000 x g and RT for 20 min. Afterward, 1 mL supernatant were centrifuged at 21 000 x g and 4°C for 1 h in a 1.5 mL polypropylene tube (Beckman Coulter, Brea, United States). The supernatant was then ultracentrifuged at 100 000 x g for 1 h at 4°C. Finally, the supernatant was discarded and sEV pellets were resuspended in 1x PBS. The protein concentration of sEV was measured via UV-Vis spectroscopy at an absorption wavelength of 280 nm to ensure constant sEV concentrations. Purified sEV were stored at 4°C and used for experiments within 48 h.

### 2.11 sEV characterization

Unpurified sEV of SK-N-AS and SK-N-SH neuroblastoma cell lines were characterized with ExoView R100 (NanoView Biosciences, Boston, United States). Cell culture supernatants were stained on tetraspanin chips (CD81, CD63, CD9, IgG control, NanoView Biosciences, Boston, United States) following the manufacturer’s instructions. In short, chips were covered with cell culture supernatants and incubated for 18 h. Then, the chips were washed, blocked and incubated with antibodies against CD81 (CF^®^555-labeled), CD63 (CF^®^647-labeled), and CD9 (CF^®^488A-labeled) (all diluted 1:600, NanoView Biosciences, Boston, United States) for 1 h. Afterward, chips were washed, dried and imaging and analysis were performed using the ExoView R100 platform. For the rest of this manuscript, the localization of multiple tetraspanins on the same sEV will be referred to as colocalization.

Purified sEV were visualized via transmission electron microscopy (TEM). For this purpose, sEV were isolated by ultracentrifugation and resuspended in 1x PBS. SEV were diluted to a protein concentration of 0.05 mg/mL in 1x PBS. The formvar carbon coated nickel grid (Plano, Wetzlar, GER) was covered with 15 µL sample at RT for 10 min. Then, the sEV were fixed on the grid for 10 min with 2% formaldehyde (FA, Carl Roth, Karlsruhe, GER) and washed 3x with Milli-Q water (MQ). All samples were imaged with a Zeiss EM109 electron microscope. Tetraspanins of purified sEV were analyzed on Western blot, as described above.

Particle concentration and size distribution of sEV were measured using the microfluidic resistive pulse sensing technology based nCS1™ Nanoparticle Analyzer (Spectradyne LLC, Signal Hill, United States). Samples of cell culture supernatants from SK-N-AS were diluted 1:10 in 1x PBS with 1% Tween 20 (Sigma-Aldrich, St. Louis, United States) and 3 µL of each sample was loaded into factory precalibrated TS-300 cartridges (Spectradyne LLC, Signal Hill, United States). The cartridges with a measurement range of 50-300 nm and the system were primed with running buffer containing 1x PBS and 1% Tween 20. Raw data were analyzed using the nCS1 software version 2.5.0.249.

### 2.12 miR-574-5p overexpression in sEV

In order to overexpress miR-574-5p in sEV, the XMIRXpress Lentivector system (System Biosciences, Palo Alto, United States) was used as previously described (Hegewald et al., 2020). A respective negative control was generated with the XMIRXP-NT system (System Biosciences, Palo Alto, United States).

SK-N-AS cells were seeded at a density of 7 × 10^5^ and A549 cells at a density of 5 × 10^5^ cells per well in a 6-well plate the day before transfection. SK-N-SH cells were seeded at a density of 1.4 × 10^5^ cells per well and 2106T cells at a density of 1 × 10^5^ cells per well in a 12-well plate. SK-N-AS and A549 cells were transfected with 2 µg plasmid and Lipofectamin 2000^®^ (Invitrogen, Karlsruhe, GER) according to the manufacturer’s instructions. SK-N-SH cells were transfected with 3 μg plasmid per well using polyethyleneimine (PEI, 1 g/L, Sigma-Aldrich, St. Louis, United States (pH 7)). In detail, 6 μl PEI were mixed with 100 μl cell culture medium without supplements. Plasmids were mixed with 100 μl DMEM without supplements and combined with the PEI mix and incubated at RT for 15 min. According to the same protocol, 2106T cells were transfected with 2 μg plasmid per well and PEI (pH 10). After 18 h, the supernatant was harvested and centrifuged at 2,000 x g and RT and stored at −80°C.

### 2.13 RNase and triton X-100 treatment of sEV

RNase and Triton X-100 treatment of sEV was performed as previously described (Donzelli et al., 2021). In brief, sEV were isolated, pooled and treated with RNase and Triton X-100. Afterward, RNA was extracted and analyzed via RT-qPCR.

### 2.14 Tetrazolium reduction assay

Cell viability was controlled using a 3-(4,5-dimethylthiazol-2-yl)-2,5-diphenyltetrazoliumbromide (MTT) tetrazolium (Carl Roth, Karlsruhe, GER) reduction assay as previously described (Donzelli et al., 2021). In brief, 1 × 10^4^ HFL1 cells per well were seeded in 96-well plates for 24 h and then stimulated with 2 μg/mL miR-574-5p oe or ScrC sEV and 10 ng/mL Transforming growth factor (TGF)-β for 72 h. Cells without sEV treatment were used as controls. Then, cells were incubated with 5 mg/mL MTT in cell culture medium for 3 h. The reaction was stopped by aspirating the medium and resuspension of the cells in 100 µL DMSO per well. The assay was evaluated using a Tecan Infinite M 200 plate reader (Tecan Group, Männedorf, CHE) by measuring the formazan quantity at 570 nm and reference at 630 nm. Reference values were subtracted from formazan measurements and sEV treated samples were normalized to samples without sEV treatment.

### 2.15 Live cell imaging

HFL1, SK-N-AS or SK-N-SH cells were seeded to 8-well chamber slides (IBIDI, Gräfeling, GER) at a density of 2.6 × 10^4^ cells per well or to a µ-24-well plate with black walls (IBIDI,Gräfeling, GER) at a density of 6 × 10^4^ cells per well. After 24 h, cell nuclei were stained with 5 μg/mL Hoechst 33258 (Sigma-Aldrich, St. Louis, United States) for 1 h at 37°C. Isolated sEV were stained with the lipophilic tracer 3,3′- dioctadecyloxacarbocy-anine perchlorate (DiO, Sigma-Aldrich, St. Louis, United States) for 15 min at 37°C. After the sEV were added, cells were imaged at 5 min intervals for a total duration of 30-60 min. Imaging was performed with an UltraVIEW VoX spinning disk system (PerkinElmer, Waltham, United States) mounted on a Nikon TI microscope (Nikon, Minato, Japan) or with a Nikon Eclipse Ti equipped with a climate chamber (37°C, 5% CO_2_, 60% humidity). Images were acquired with a cooled 14-bit EMCCD camera (1,000 × 1,000-pixel frame transfer EMCCD, 30 fps at full frame 1 × 1 binning 35 MHz readout, 8 × 8 μm pixel size) using Velocity 6.3 (PerkinElmer, Waltham, United States) or a Nikon DS-Qi2 camera and NIS elements software (Nikon, Minato, Japan). To observe the uptake of sEV via endocytosis, HFL1 cells were incubated with 20 μg/mL pHrodo™ Red Dextran (Invitrogen, Karlsruhe, GER) together with DiO-labeled sEV. After 30 min–1 h, cells were washed briefly with 1x PBS and fresh cell culture medium was added. Cells were imaged within 15 min.

### 2.16 Migration assay

Cells were seeded in a 12-well plate at a density of 1 × 10^5^ cells per well. After 24 h, cells were scratched in a cross shape twice in every well. The medium was aspirated, and the cells were washed gently 3 times with starvation medium without FCS. Then, cells were stimulated with 2 μg/mL sEV, 10 ng/mL TGF-β or 10 ng/mL R848. Immediately after the stimulation, images of each cross were taken with an Axio Vert.A1 and the ZEN 2011 software. The cells were then allowed to migrate in the incubator at 37°C and 5% CO_2_ for 13 h. After 13 h, cells were washed briefly in 1x PBS and fixated with 3.7% FA in 1x PBS for 10 min at RT. Cells were washed again briefly with 1x PBS and stained with hematoxylin solution (Sigma-Aldrich, St. Louis, United States) for 5 min at RT. Finally, cells were washed 3 × 10 min with 1x PBS and final images of the crosses were taken with the Axio Vert.A1. Brightness and contrast of each image were optimized with the ImageJ Software (https://imagej.nih.gov/ij/) and migrated cells were counted with the cell counter plugin (https://imagej.nih.gov/ij/plugins/cell-counter.html). At the same time, the cells surrounding the scratch were also counted, and migrated cells were normalized to non-migrated cells. The relative cell migration between comparable scratches was then normalized to cells stimulated with ScrC sEV.

### 2.17 Antibody blocking of sEV

Cells were seeded in 12-well plates at a density of 1 × 10^5^ cells per well 24 h prior to stimulation. MiR-574-5p oe sEV were isolated from the supernatant of A549 or SK-N-AS cells and resuspended in 1x PBS. 1 μg sEV were incubated with 100 ng antibody against CD81 (α-CD81, MABF 2061, Sigma-Aldrich, St. Louis, United States), CD63 (α-CD63, NBP2-42225, Novus Biologicals, Littleton, GER), CD9 (α-CD9, sc-59140, Santa Cruz Biotechnology, Dallas, United States), or Mouse IgG1 Isotype Control (MAB002, Novus Biologicals, Littleton, United States) in a total volume of 20 µL. Appropriate antibody concentrations were tested previously. All reactions were incubated at 4°C for 21 h. Then the previously seeded cells were washed briefly with 1x PBS and the medium was switched to sEV-depleted medium. Cells were stimulated with sEV previously blocked with antibodies or with antibodies without sEV for 24 h at 37°C. Then, cells were harvested, and Western blot analysis was performed.

### 2.18 Luciferase-assay

SEV-donor cells SK-N-AS and A549 were seeded at a density of 5 × 10^5^ cells in a 6-well plate the day before transfection. Then, cells were transfected with 2 µg Nano-Luciferase (NLuc)-Hsp-70 plasmid (Bonsergent et al., 2021) and Lipofectamin 2000^®^ according to the manufacturer’s instructions 18 h before sEV harvesting. SEV were harvested from the supernatant of A549 or SK-N-AS cells. 2 μg sEV were blocked with 200 ng as previously described in a total volume of 40 µL for 21 h. Acceptor cells were seeded in 96-well plates at a density of 1.5 × 10^4^ cells per well in quadruplets 24 h prior to stimulation. Cells were stimulated with 0.5 µg blocked sEV for 4 h at 37°C and Luciferase-assay was performed using Nano-Glo^®^ Luciferase (Promega, Madison, United States) according to the manufacturer`s instructions.

### 2.19 sEV uptake quantification by confocal microscopy

A549 or HFL1 cells were seeded in 8-well cell culture slides (SPL life sciences, Naechon-myeon, KOR) at a density of 1.2 × 10^4^ cells per well. As described above, sEV derived from A549 or SK-N-AS cells were isolated and blocked with antibodies. Then, sEV were stained with DiO for 30 min and dialyzed against 1x PBS using a 14 kDa membrane for 1 h. The previously seeded cells were washed briefly with 1x PBS, and the medium was switched to sEV-depleted medium containing stained sEV. After 4 h, cells were washed 2 x with 1x PBS and fixed with 3.7% FA for 15 min. Cells were washed with 1x PBS 2 x and then incubated for 45 min in a staining solution (1% BSA; 1 μg/mL 4′,6-Diamidino-2-phenylindol (DAPI); Phalloidin i647,1:1000, Abcam, Cambridge, United Kingdom). Then, cells were washed 2 x with 1x PBS, 1 x with MQ, and mounted with a glass coverslip using Mowiol-488. Stained cells were visualized with a confocal Leica DMi8 microscope. The images were analyzed with a custom ImageJ script. First, the 3D multichannel microscope images were 2D max Z-projected and the dimensions of the cell to be explored were determined using an automatic intensity threshold based on the actin skeleton. The captured sEV within the cell were detected and counted using a classifier trained with the WEKA segmentation algorithm (doi:1093/bioinformatics/btx180). The number of sEV was normalized to the cell area to determine the relative uptake rate.

### 2.20 Statistics

All results are shown as mean + standard error of mean (SEM) of at least three independent experiments. Statistical analysis was carried out by Student’s unpaired t-test (two-tailed) or one-way ANOVA with Tukey’s multiple comparison test using GraphPad Prism 9.0. Experimental differences were considered as significant for *p* ≤ 0.05 (indicated as * for *p* ≤ 0.05, ** for *p* ≤ 0.01, *** for *p* ≤ 0.001, and **** for *p* ≤ 0.0001, or § for *p* ≤ 0.05, §§ for *p* ≤ 0.01, §§§ for *p* ≤ 0.001, and §§§§ for *p* ≤ 0.0001).

## 3 Results

### 3.1 mPGES-1, CUGBP1 and miR-574-5p colocalize in 11q-deleted neuroblastoma

**FIGURE 1:**
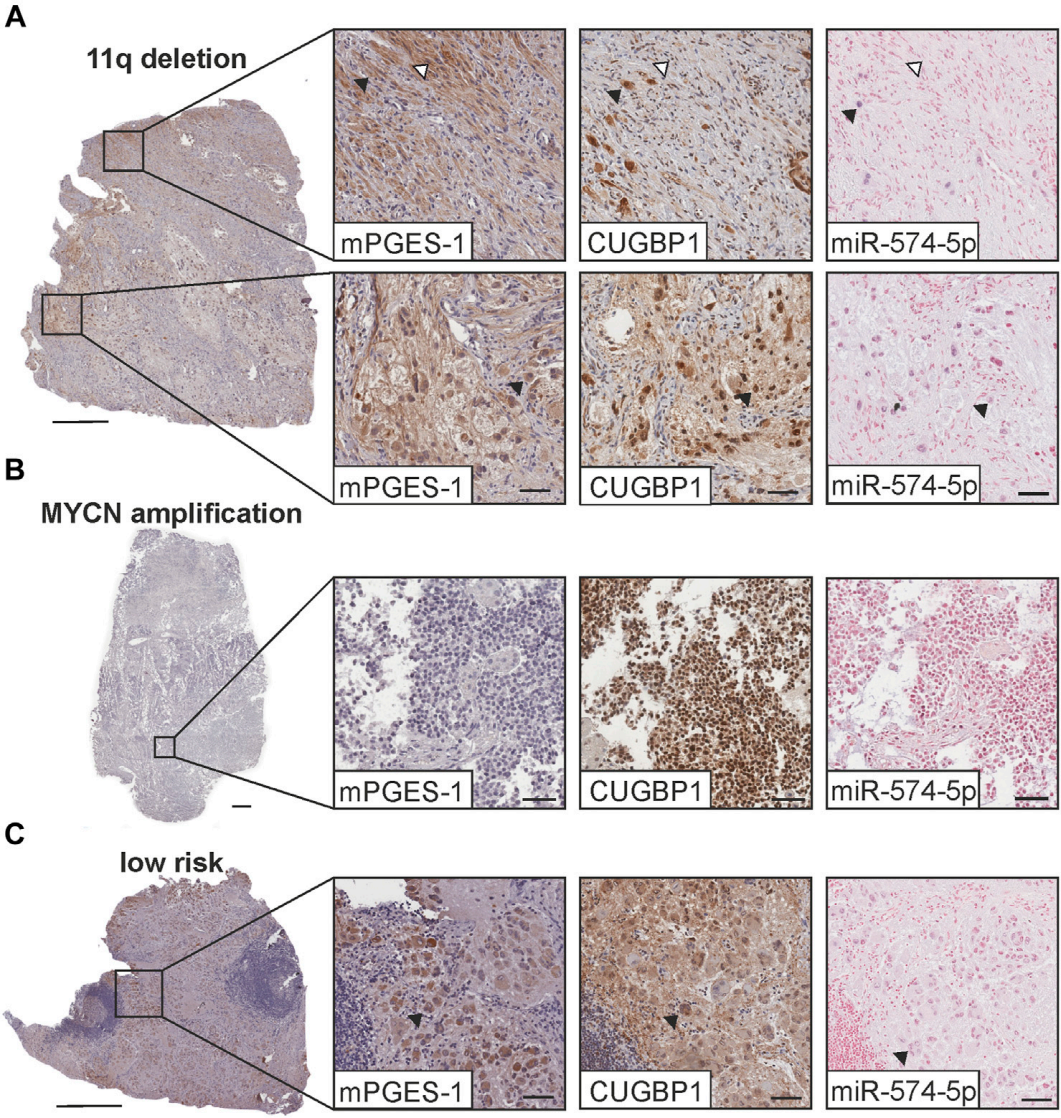
MPGES-1 and CUGBP1 immunostaining (IHC) and *in situ* hybridization (ISH) of miR-574-5p in neuroblastoma tumor sections. IHC (brown) was counterstained with hematoxylin (blue). ISH was performed using a miR-574-5p-probe (blue) and sections were then counterstained with nuclear fast red (red). Exemplary tumor sections of neuroblastoma tumor subtypes with **(A)** 11q deletion, **(B)** MYC-N amplification and **(C)** of the low-risk subtype. Fibroblasts in the 11q deleted subtype (white arrows) are positive for mPGES-1 and differentiated tumor cells (black arrows) positive for mPGES-1, CUGBP1 and miR-574-5p. Scale bars: 0.5 mm, magnified images: 50 µm.

The interaction between miR-574-5p and CUGBP1 regulates PGE_2_ biosynthesis in NSCLC (Saul et al., 2019b; Emmerich et al., 2020). To determine whether this novel PGE_2_ regulatory mechanism is also present in neuroblastoma, we performed immunostaining on serial sections for mPGES-1 and CUGBP1 as well as ISH with specific miR-574-5p complementary locked nucleic acid probes. We analyzed samples from 20 patients with different neuroblastoma subtypes (11q-deleted and MYCN Proto-Oncogene (*MYCN)*-amplified high-risk tumors, and low-risk). Several risk factors play a role in the classification of neuroblastoma (Cohn et al., 2009). With both genetic mutations deletion within chromosome 11q and amplification of the *MYCN* oncogene, the chance of cure decreases significantly (Mlakar et al., 2017). In 11q-deleted tumors (Figure 1A), we found fibroblasts positive for mPGES-1 (white arrows), consistent with observations by Larsson et al. (Larsson et al., 2015). However, these cells were negative for miR-574-5p. In *MYCN*-amplified tumors (Figure 1B), no cells in the TME were positive for mPGES-1 or miR-574-5p. Interestingly, we observed cancer cells co-expressing mPGES-1, CUGBP1, and miR-574-5p in the 11q-deleted and low-risk tumors (black arrows) (Figures 1A, C, negative controls in Supplementary Figure S1). Based on their histological appearance, we identified these cells as differentiated neuroblastoma cells, presumably ganglion cells (Hicks and Mackay, 1995). Strikingly, miR-574-5p and CUGBP1 expression were detected in the nuclei of these cells, characteristic of the miR-574-5p/CUGBP1 decoy mechanism that regulates alternative splicing of mPGES-1 3′UTR (Saul et al., 2019b).

**FIGURE 2:**
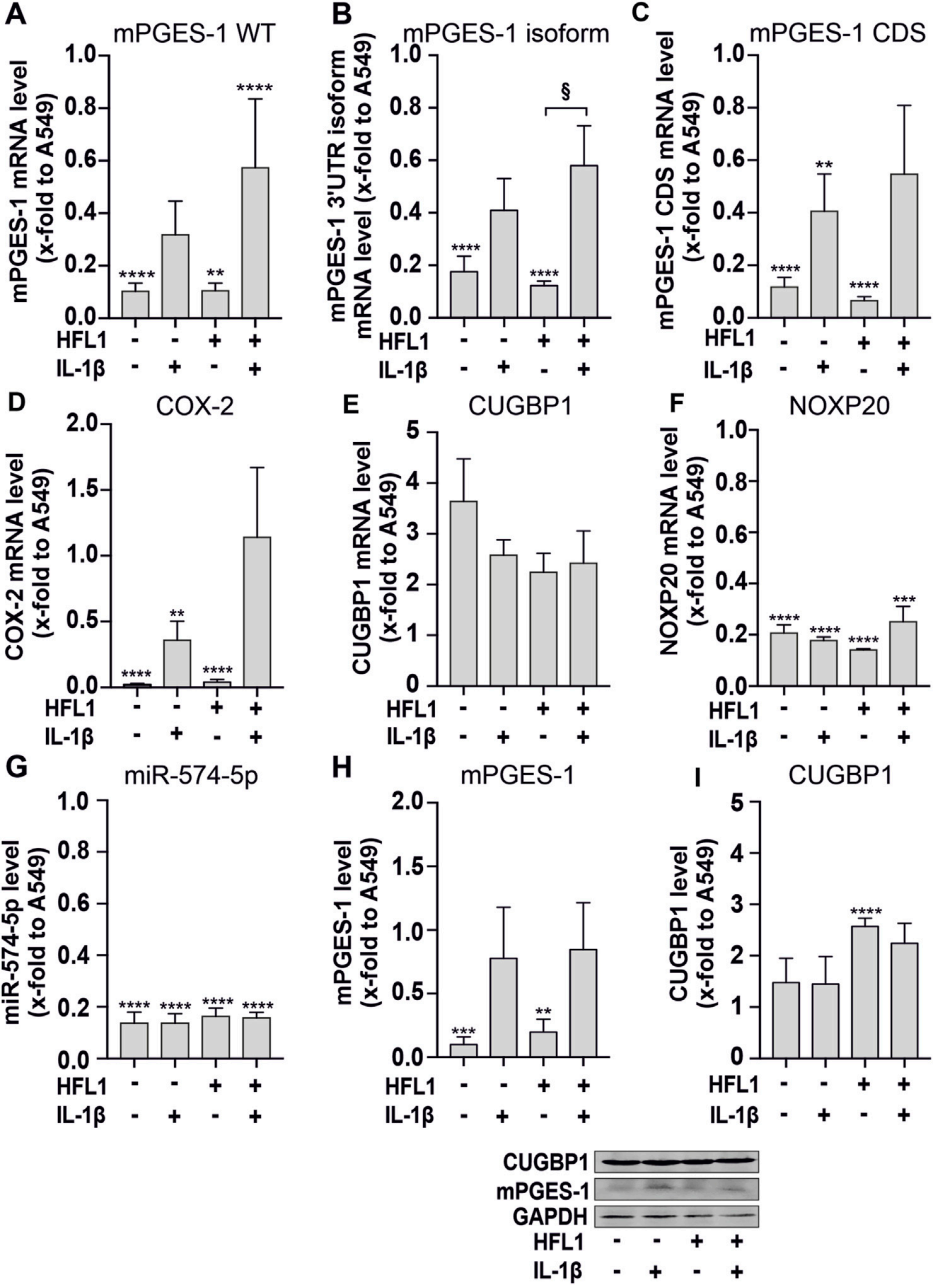
3D-cell culture experiments of SK-N-AS cells combined with human lung fibroblasts (HFL1). **(A–G)** RT-qPCR analysis of mPGES-1 wild type (WT), mPGES-1 3′untranslated region (UTR) isoform, mPGES-1 coding sequence (CDS), cyclooxygenase (COX)-2, CUGBP1, the host gene of miR-574-5p NOXP20, and miR-574-5p RNA. Mono- and coculture spheroids of SK-N-AS cells combined with HFL1 were cultured for 72 h and then stimulated with 5 ng/mL interleukin (IL)-1β for 24 h. MRNA levels were normalized to GAPDH and miR-574-5p levels to spike-in control ath-miR-159a and untreated A549 monoculture spheroids to compare the amount of mRNA between the cell lines. Data are presented as mean +SEM (N = 4). **(H,I)** Western blot analysis of mPGES-1 and CUGBP1. Protein levels were normalized to GAPDH and A549 monocultures. Data are presented as mean + SEM (N = 4). Unpaired t-test to untreated A549 monocultures, *p ≤ 0.05; **p ≤ 0.01; ***p ≤ 0.001; ****p ≤ 0.0001. One-way ANOVA to other samples § *p* ≤ 0.05.

We therefore investigated the decoy mechanism and regulation of mPGES-1-dependent PGE_2_ synthesis in the 11q-deleted neuroblastoma SK-N-AS cells. For this purpose, the relative expression of mPGES-1, CUGBP1 and miR-574-5p was determined in SK-N-AS spheroids in comparison to A549 spheroids (Figure 2). A549 cells were used as a model system because the miR-574-5p-mediated regulation of PGE_2_ in this cell system has been well characterized both *in vitro* and *in vivo*. In our experimental set-up, we have co-cultured the cancer cells with fibroblasts. Fibroblasts play a critical role in tumorigenesis in NSCLC and neuroblastoma (DuBois et al., 1999; Kock et al., 2018). We used the lung fibroblast cell line HFL1 for our co-culture experiments because neuroblastoma metastasizes to the lung and other sites (Maman et al., 2013). This allowed us to analyze the influence of both tumor types on fibroblasts derived from the same tissue. It also provided a basis for comparison of our results with those obtained with NSCLC. The spheroids were also treated with IL-1β to create a pro-inflammatory environment.

Our 3D cell culture experiments with SK-N-AS cells confirmed the tissue staining results. In general, the expression of mPGES-1 on both mRNA and protein levels was significantly lower in neuroblastoma than in NSCLC cells. Thus, we detected significantly lower RNA expression of mPGES-1 and COX-2 in both mono- and co-cultured SK-N-AS spheroids compared to A549 spheroids (Figures 2A–D). In the presence of the miR-574-5p/CUGBP1 decoy mechanism, alternative splicing produces the mPGES-1 3′-UTR isoform, which has a higher translation rate than WT mPGES-1 mRNA (Saul et al., 2019b). We detected the 3′-UTR isoform of mPGES-1 for the first time in SK-N-AS spheroids (Figure 2B). IL-1β stimulation induced mPGES-1, its isoform and COX-2 mRNA levels as well as mPGES-1 protein levels (Figure 2H).

It is interesting to note that miR-574-5p levels were significantly lower in SK-N-AS spheroids than in A549 spheroids. They were not affected by IL-1β stimulation (Figure 2G). This is consistent with the low mRNA expression of NOXP20, which carries the miR-574-5p precursor in an intron (Figure 2F). In contrast, increased levels of CUGBP1 were detected in SK-N-AS spheroids compared to A549 spheroids (Figure 2I). This suggests that CUGBP1 controls the expression of mPGES-1 by binding to miR-574-5p.

Finally, we performed RNA immunoprecipitation of CUGBP1 to demonstrate that the miR-574-5p/CUGBP1 decoy mechanism regulates the expression of mPGES-1 in SK-N-AS cells (Supplementary Figure S2). We showed that both miR-574-5p and mPGES-1 mRNA together with CUGBP1 were highly enriched in both untreated and IL-1ß stimulated cells.

Taken together, our results demonstrate that miR-574-5p and CUGBP1 were involved in the regulation of mPGES-1 expression and PGE_2_ synthesis in neuroblastoma. However, this occurs to a lesser extent compared to NSCLC.

### 3.2 PGE_2_ induces secretion of miR-574-5p in sEV via EP1/3 binding in SK-N-AS cells

**FIGURE 3:**
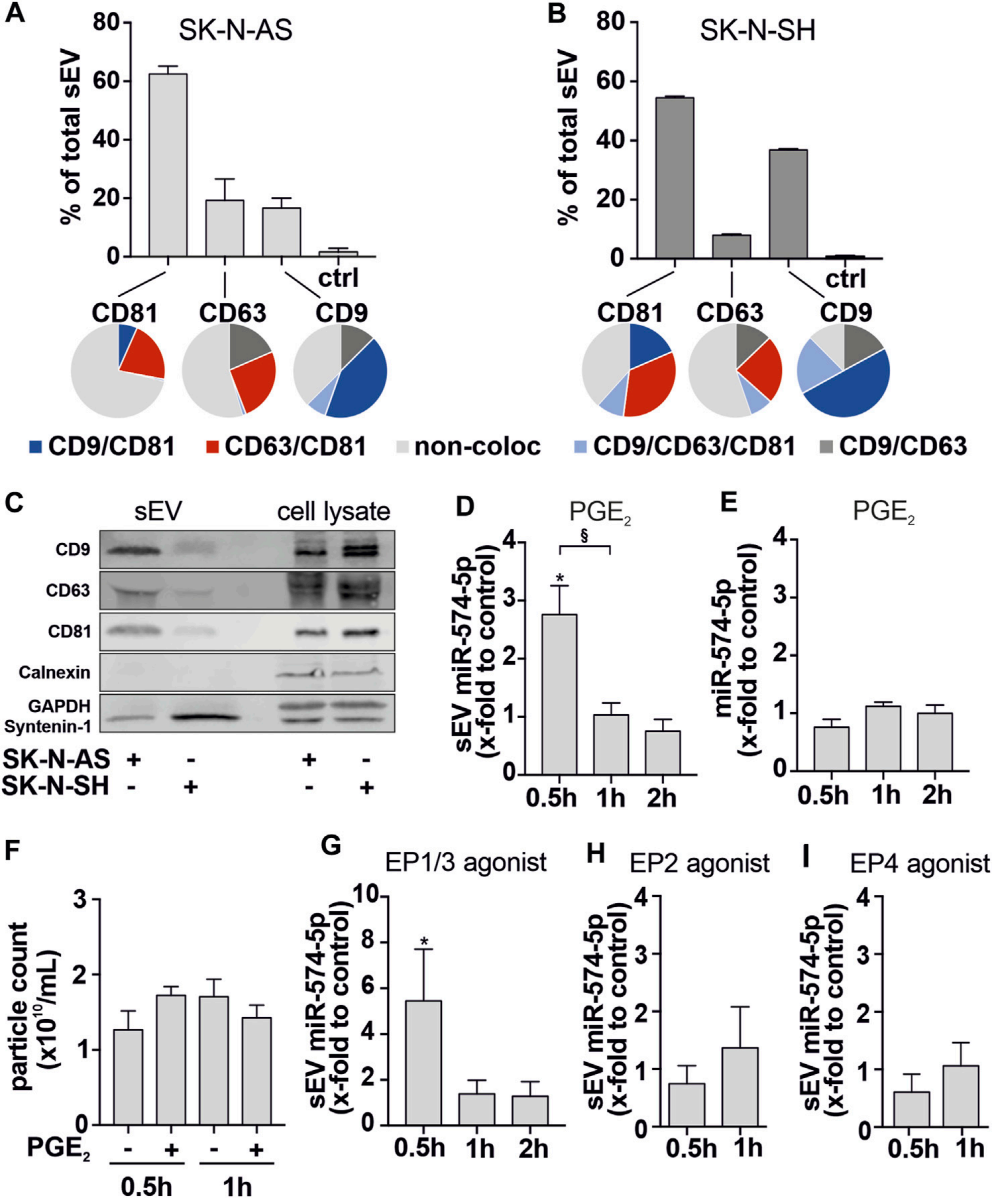
Characterization of sEV derived from SK-N-AS and SK-N-SH cells. **(A, B)** Unpurified SK-N-AS- and SK-N-SH-derived sEV were analyzed using the ExoView R100 platform (NanoView Biosciences). SEV were captured at specific antibody-coated spots against CD81, CD63, and CD9 (bar graphs). Captured sEV were further analyzed for tetraspanin colocalization with specific fluorescent antibodies (pie charts) **(C)** Western blot analysis of tetraspanins CD9, CD63,CD81, Calnexin, Syntenin-1 and GAPDH of sEV and cell lysates from SK-N-AS and SK-N-SH cells. A representative blot of 3 independent experiments is shown and **(D)** sEV-miR-574-5p level and **(E)** intracellular miR-574-5p level of SK-N-AS cell supernatants and cells. Cells were stimulated with 5 nM PGE_2_ for 0.5 h, 1 h and 2 h prior to supernatant harvesting. **(F)** Particle count measured using a nCS1™ Nanoparticle Analyzer showed no significant effect on SK-N-AS particle numbers secreted after PGE_2_ treatment. For further analysis, cells were stimulated with 5 nM **(G)** PGE_2_ receptor (EP)1/3 agonist Sulprostone, **(H)** EP2 agonist Butaprost and **(I)** EP4 agonist L-902,688 or respective control solvents. MiR levels were analyzed by RT-qPCR, normalized to the spike-in control ath-miR-159a and folded to their corresponding control. Data are shown as mean + SEM (N = 3-4). Unpaired t-test to corresponding control *p ≤ 0.05. Unpaired t-test to other samples, § *p* ≤ 0.05.

Our next step was to validate whether neuroblastoma cells are also able to specifically sort miR-574-5p into sEV upon stimulation by PGE_2_ like NSCLC cell lines (Donzelli et al., 2021). We included another high-risk neuroblastoma cell line, SK-N-SH, without 11q deletion or *MYCN* amplification, as a complementary model to the SK-N-AS cell line to monitor possible neuroblastoma-specific differences. Therefore, SK-N-AS and SK-N-SH cells were stimulated with 5 nM PGE_2_. After 30 min, 1 h and 2 h we purified sEV from cell culture supernatants by differential ultracentrifugation and characterized neuroblastoma sEV populations by TEM (Supplementary Figure S3A), ExoView R100 (Figures 3A, B; Supplementary Figures S3B, C) and Western blot analysis (Figure 3C). Total RNA was isolated from both sEV and neuroblastoma cells for RT-qPCR analysis of extracellular and intracellular miR-574-5p levels.

We observed that sEV-derived miR-574-5p was significantly upregulated after 30 min of PGE_2_ stimulation and strongly decreased after 1 h (Figure 3D). However, the intracellular level of miR-574-5p (Figure 3E) and particle number (Figure 3F) were not affected by PGE_2_ stimulation. Notably, the secretion effect was mediated by PGE_2_ receptors EP1/3 receptors (Figures 3G–I). PGE_2_ stimulation of SK-N-SH cells induced miR-574-5p secretion 2 h after treatment (Supplementary Figure S3D). Intracellular miR-574-5p levels were unaffected (Supplementary Figure S3E).

Taken together, these data suggest that the stimulation of EP1/3 receptors by PGE_2_ induces the secretion of sEV-miR-574-5p in neuroblastoma cell lines.

### 3.3 sEV populations derived from neuroblastoma cell lines differ in their tetraspanin composition

The sEV envelope consists of a lipid bilayer in which a large number of membrane proteins are embedded (Thery et al., 2002). A highly enriched family of membrane proteins on sEVs are the tetraspanins CD9, CD63, and CD81, which are used as classical sEV markers (Andreu and Yáñez-Mó, 2014; Kowal et al., 2016). The composition of these sEV markers can be used to characterize the sEV population of cells from different origins (Breitwieser et al., 2022). Therefore, we used the ExoView R100 platform to characterize the sEV populations of the neuroblastoma cell lines SK-N-AS and SK-N-SH using these markers at the single vesicle level.

We observed that SK-N-AS- and SK-N-SH-derived sEV had similarly high levels of CD81 (Figures 3A, B). SK-N-AS-derived sEV had the lowest number of sEV bound to CD9 and CD63 spots, each accounting for approximately 20% of total sEV. In contrast, almost 40% of the total SK-N-SH-derived sEV were bound to the CD9 spot.

Next, we analyzed the localization of CD81, CD63, and CD9 on the same sEV (Figures 3A, B, pie charts). While CD63 and CD9 positive sEV showed very similar colocalization patterns in both cell types, CD81 positive sEV revealed cell-specific differences. In 7% of SK-N-AS-derived CD81 positive sEV, CD81 and CD9 colocalized. 21% showed a colocalization of CD81 and CD63, while less than 1% of sEV were positive for all three tetraspanins. Of note, 71% of CD81 positive SK-N-AS-derived sEV did not colocalize with other tetraspanins. In contrast, in SK-N-SH-derived sEV, only 38% were single positive for CD81.19% of CD81-positive SK-N-SH-derived sEV showed colocalization of CD81 and CD9, while 35% were positive for CD81 and CD63. Here, 10% of all CD81-positive derived sEV were positive for all three tetraspanins. These results show that SK-N-AS- and SK-N-SH-derived sEV have different tetraspanin compositions. These might influence the uptake of the sEV by possible target cells. Finally, we determined the distribution of the particle size of SK-N-AS- and SK-N-SH-derived sEV via light scattering. The particle size peaked at 60 nm for both cell lines (Supplementary Figures S3B, C). TEM analysis confirmed the diameter of sEV to be 50–150 nm (Supplementary Figure S3A), which is consistent with our findings from the ExoView R100 analysis.

In conclusion, the sEV populations of the neuroblastoma cell lines exhibited a unique tetraspanin composition. This suggests that neuroblastoma-specific sEV may specifically interact with target cells in the tumor environment.

### 3.4 SK-N-AS sEV-derived miR-574-5p increases α-SMA levels in HFL1 cells via TLR7/8

**FIGURE 4:**
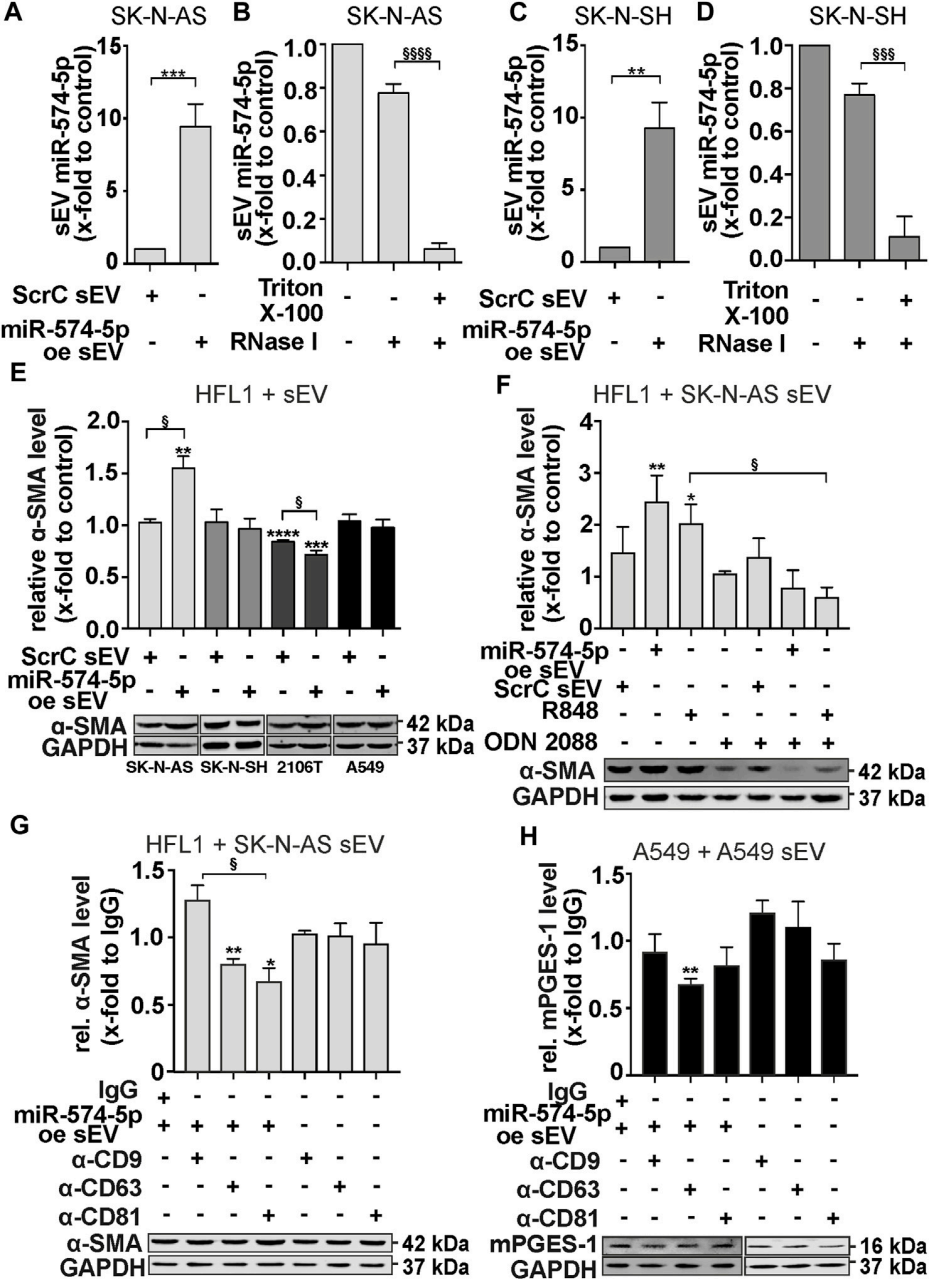
sEV-miR-574-5p derived from SK-N-AS cells induce α-SMA level of HFL1 cells via TLR7/8. **(A, C)** sEV with miR-574-5p overexpression (oe) of SK-N-AS and SK-N-SH cells were generated using XMIR-Xpress plasmids for miR-574-5p and scrambled control (ScrC). MiR-574-5p levels were analyzed via RT-qPCR, normalized to the spike-in control ath-miR-159a and folded to their corresponding negative control (N = 3). Relative changes to ScrC are shown as mean + SEM, unpaired t-test **p ≤ 0.01; ***p ≤ 0.001. **(B, D)** MiR-574-5p oe sEV of SK-N-AS and SK-N-SH were treated with Triton X-100 and RNase **(I)**. In samples without Triton X-100, miR-574-5p was protected from RNase digest. MiR-574-5p levels were analyzed via RT-qPCR, normalized to the spike-in control ath-miR-159a and folded to their corresponding negative control (SK-N-AS: N = 6, SK-N-SH: N = 5). Relative changes are shown as mean + SEM, unpaired t-test to samples without Triton X-100 §§§*p* ≤ 0.001; §§§§*p* ≤ 0.0001. **(E)** Western blot analysis of α-SMA protein levels in HFL1 cells treated with TGF-β and 2 μg/mL miR-574-5p oe or ScrC sEV derived from neuroblastoma cell lines SK-N-AS and SK-N-SH and lung cancer cell lines A549 and 2106T for 72 h α-SMA levels were normalized to GAPDH and folded to untreated cell samples (SK-N-AS, A549: N = 3, SK-N-SH, 2106T: N = 4). **(F)** Western blot analysis of α-SMA protein levels in HFL1 cells treated with SK-N-AS-derived miR-574-5p oe or ScrC sEV. HFL1 cells were treated with 2 μg/mL sEV, 100 ng/mL R848 (TLR7/8 ligand) or 200 mM ODN 2088 Control (ODN 2087) (TLR7/8 antagonist) for 72 h α-SMA levels were normalized to GAPDH and folded to untreated cell samples (N = 4). **(G)** Western blot analysis of α-SMA protein levels in HFL1 cells treated with 2 μg/mL miR-574-5p oe sEV of SK-N-AS previously blocked with α-CD9, α-CD63, α-CD81 or mouse IgG antibodies or antibodies without sEV for 21 h. Cells were treated for 24 h α-SMA levels were normalized to GAPDH and folded to IgG control samples (N = 3-4). **(H)** Western blot analysis of mPGES-1 protein levels in A549 cells treated with 2 μg/mL miR-574-5p oe sEV of A549 cells previously blocked with α-CD9, α-CD63, α-CD81 or mouse IgG antibodies or antibodies without sEV for 21 h. Cells were treated for 24 h mPGES-1-levels were normalized to GAPDH and folded to IgG control samples (N = 3-4). All Western blot results are shown as mean + SEM, unpaired t-test to untreated control or IgG control, *p ≤ 0.05; **p ≤ 0.01; ***p ≤ 0.001; ****p ≤ 0.0001; unpaired t-test to other samples, § *p* ≤ 0.05.

To analyze the physiological function of sEV-derived miR-574-5p in neuroblastoma, we established an overexpression (oe) system for SK-N-AS and SK-N-SH cell lines (Figures 4A, C). The system enhanced miR-574-5p loading into sEV (miR-574-5p oe sEV). Control experiments were performed with sEV loaded with a scrambled miR (ScrC sEV). We compared miR-574-5p levels of both sEV types by RT-qPCR and detected a ∼9-fold increase of miR-574-5p in both SK-N-AS-derived oe sEV and SK-N-SH-derived oe sEV (Figures 4A, C). To verify whether miR-574-5p was loaded into sEV and not attached to the outside, we performed an RNase protection experiment with SK-N-AS and SK-N-SH-derived miR-574-5p oe sEV. sEV were treated with RNase alone or combined with detergent followed by RT-qPCR analysis (Figures 4B, D). We demonstrated that the majority of miR-574-5p was protected within sEV with engineered miR-574-5p levels. The miR-574-5p levels were significantly decreased by disruption of sEV membrane followed by RNase digestion. This suggests that most of the miRs are located within sEV, as miR-574-5p was protected in samples without detergent.

Next, we stimulated SK-N-AS cells with engineered sEV derived from SK-N-AS cells and analyzed the levels of mPGES-1 and COX-2. As in A549 cells, we hypothesized that mPGES-1 levels would be reduced by sEV-derived miR-574-5p (Donzelli et al., 2021). No effects on mPGES-1 protein and mRNA levels were observed in response to miR-574-5p oe sEV or with the TLR7/8 ligand R848 (Supplementary Figures S4A, B). Notably, we detected mPGES-1 protein levels only after stimulation with IL-1β. This indicated that sEV-derived miR-574-5p did not have an autocrine function on mPGES-1-dependent PGE_2_ biosynthesis in SK-N-AS cells. Therefore, we hypothesized that sEV-derived miR-574-5p may act in a paracrine manner in the neuroblastoma tumor environment. Since fibroblasts play a critical role in the tumor progression of neuroblastoma (Kock et al., 2018; Kock et al., 2020), we aimed to analyze the physiological function of sEV-derived miR-574-5p on fibroblasts.

Our first question was whether fibroblasts internalize sEV from neuroblastoma cells. This was confirmed by a live cell microscopy experiment using HFL1 cells (Supplementary Video S1). Next, we treated the HFL1 cells with miR-574-5p oe sEV or ScrC sEV derived from the neuroblastoma cell lines SK-N-AS and SK-N-SH and the lung cancer cell lines A549 and 2106T, combined with and without TGF-β stimulation. The additional stimulation of TGF-β was used to enhance the differentiation process of the fibroblasts (Frangogiannis, 2020). Western blot analysis showed that the level of α-SMA was slightly increased after treatment with miR-574-5p oe sEV derived from SK-N-AS (Supplementary Figure S4C). Additional stimulation with TGF-β resulted in an even more significant 1.5-fold increase after stimulation with SK-N-AS-derived miR-574-5p oe sEV (Figure 4E). This effect on α-SMA levels was not observed with SK-N-SH, 2106T, and A549-derived miR-574-5p oe sEV. The effect of SK-N-AS-derived sEV could not be attributed to increased fibroblast proliferation (Supplementary Figure S4D). In addition, initial live cell microscopy experiments revealed different uptake patterns of SK-N-AS and SK-N-SH-derived sEV by their cells, suggesting an influence of uptake on sEV function (Supplementary Figures S4F, G).

Since miR-574-5p can activate TLR7/8 signaling (Fabbri et al., 2012; Hegewald et al., 2020), we included the TLR7/8 ligand R848 as a positive control and the inhibitor ODN 2087 in our experiments (Hackstein et al., 2011; Römmler et al., 2015). The α-SMA level was increased by the addition of miR-574-5p oe sEV and R848, respectively. This effect was abolished by ODN 2087, suggesting a TLR7/8-mediated effect on α-SMA in HLF1 cells (Figure 4F). Interestingly, the α-SMA mRNA level was not affected (Supplementary Figure S4E), suggesting that TLR7/8 activation influences the regulation of α-SMA at the post-transcriptional level. Notably, miR-574-5p oe sEV not only increased α-SMA levels, but also significantly increased the migratory ability of fibroblasts (Supplementary Figures S5A, B), which was independent to TLR7/8 signaling.

Taken together, our results showed a cell- and cancer-specific response to sEV-derived miR-574-5p. miR-574-5p oe sEV derived from SK-N-AS cells induced fibroblast differentiation via TLR7/8 signaling. This effect was not observed with miR-574-5p oe sEV derived from SK-N-SH or NSCLC cell lines A549 and 2106T. Interestingly, miR-574-5p oe sEV derived from SK-N-AS and SK-N-SH cells did not affect mPGES-1 expression in cancer cells, as was observed with sEV derived from A549 cells (Donzelli et al., 2021).

### 3.5 CD9, CD63 and CD81 composition influences the functionality of sEV-derived miR-574-5p

We recognized that the sEV populations secreted by the different cancer cells revealed a cell type-specific composition of tetraspanins on the sEV surface, which could have an impact on the internalization and function of the extracellular miR-574-5p. To find out whether the surface proteins CD9, CD63 and CD81 might have a decisive influence on the cell-specific effect of extracellular miR-574-5p, we blocked tetraspanins on the surface of sEV with antibodies against CD81, CD63 and CD9 and analyzed α-SMA levels in HFL1 cells. Such a similar experimental approach has been described previously (Spenlehauer et al., 2001; Sims et al., 2017). Our experiments showed that treatment of SK-N-AS-derived miR-574-5p oe sEV with antibodies against CD81 and CD63, but not CD9, significantly decreased α-SMA levels in HFL1 cells (Figure 4G). We performed the same experiment with A549-derived miR-574-5p oe sEV and found no effect (Supplementary Figure S5C). In addition to this experimental setup, we treated A549 cells with A549-derived miR-574-5p oe sEV and measured mPGES-1 levels to analyze the effect of tetraspanin blocking on the physiological function of A549-derived sEV-miR-574-5p (Figure 4H). CD63 blockade significantly decreased mPGES-1 levels in A549 cells, CD81 blockade had no effect.

**FIGURE 5:**
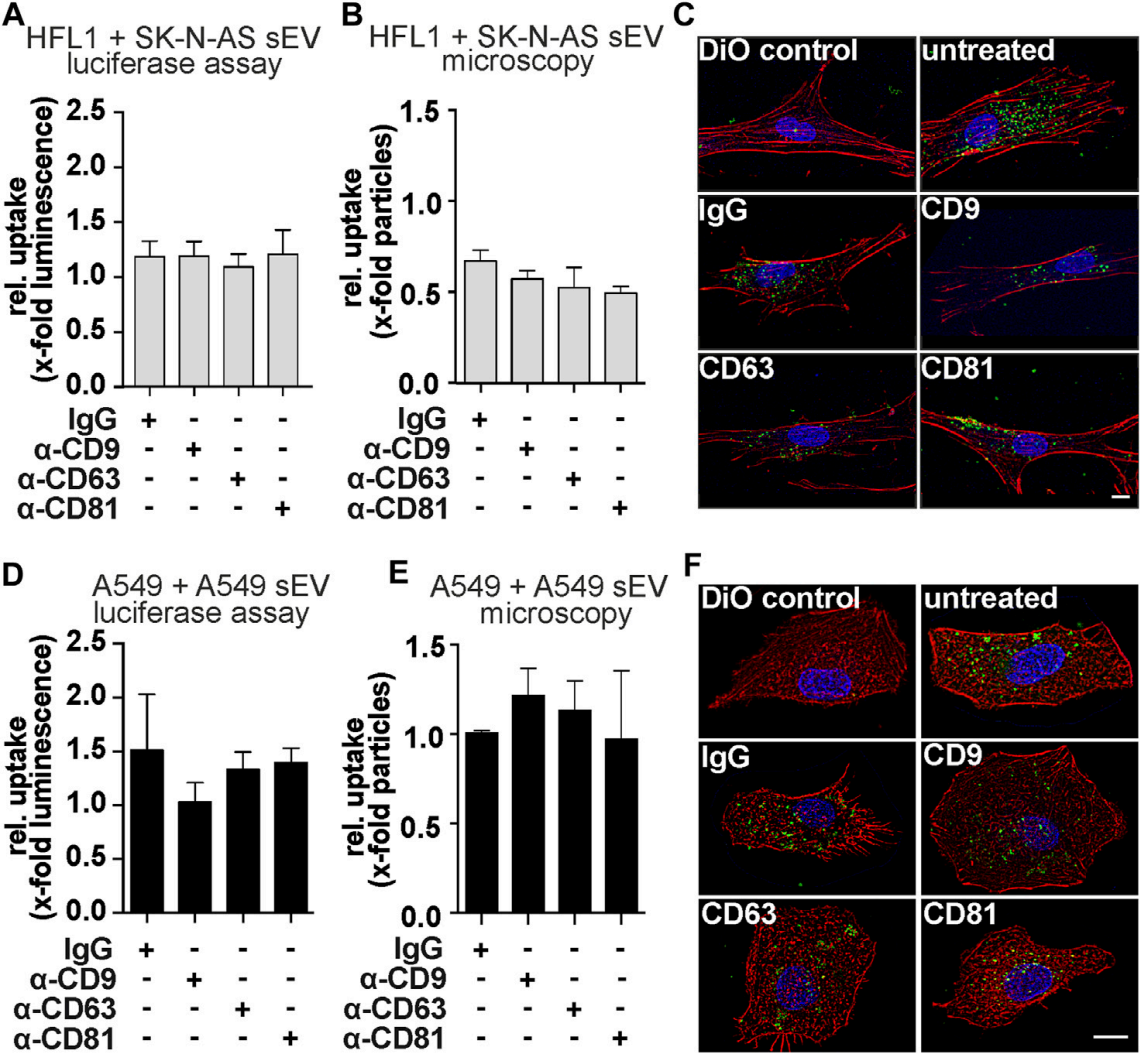
Uptake experiments of tetraspanin-blocked sEV from SK-N-AS or A549 cells. **(A)** Luciferase-assays and **(B, C)** microscopic uptake quantification of HFL1-cells treated with sEV from SK-N-AS cells. Representative micrographs were shown after 4 h of SK-N-AS sEV uptake. Scale bars = 10 µm. **(D)** Luciferase-assays and **(E, F)** microscopic uptake quantification of A549-cells treated with sEV from A549-cells. Representative A549 micrographs were shown after 4 h of A549 sEV uptake. Scale bars = 10 µm. For Luciferase-assays sEV-donor cells were transfected with NLuc-Hsp70 plasmid 18 h prior sEV-harvesting. Then, sEV were harvested and blocked with antibodies α-CD9, α-CD63, α-CD81, or mouse IgG for 21 h and afterward, luciferase assay was performed. Results are shown as mean + SEM (N = 6). For microscopy experiments, acceptor cells were exposed to blocked and DiO labeled sEV for 4 h prior to fixation. Results are normalized to unblocked sEV and depicted as the mean + SEM (N = 3).

To determine whether blockade of tetraspanins alters the rate of vesicle internalization, we used several approaches to quantify the rate of internalization. First, we used a luciferase assay (Bonsergent et al., 2021). For this, we transfected donor cells with a nano-luciferase (NLuc)-Hsp70 (a generic EV cargo) plasmid (Gao et al., 2015), isolated sEV from the supernatant, and used them in internalization experiments. Although blocking the tetraspanins CD63 and CD81 showed an effect on the function of SK-N-AS-derived sEV, we did not observe differences in internalization rates (Figure 5A). In addition, we performed a microscopy-based analysis of sEV uptake. In this assay, the blocked sEV were additionally stained with the lipophilic tracer dye DiO. After incubation of the acceptor cells with the stained sEV, the cells were fixed with formaldehyde, and the DiO speckles within were quantified. No specific changes in sEV uptake rates were observed (Figures 5B, C). Of note, the addition of antibodies (including IgG control) to SK-N-AS derived sEV resulted in a non-specific reduction of uptake by HFL1 cells in this assay. However, when CD63 was blocked on sEV derived from A549 cells, mPGES-1 protein levels were reduced in A549 cells (Figure 4H), but no significant changes in uptake rates were measured by luciferase assay (Figure 5D) or microscopy-based assay (Figures 5E, F). The fact that tetraspanins have no effect on sEV uptake rate was also confirmed by live cell microscopy experiments (Supplementary Figures S6, S7).

Taken together, these results demonstrate that blocking tetraspanins at the sEV envelope modulates the functionality of sEV-derived miR-574-5p without altering the overall vesicle uptake rate.

## 4 Discussion

**FIGURE 6:**
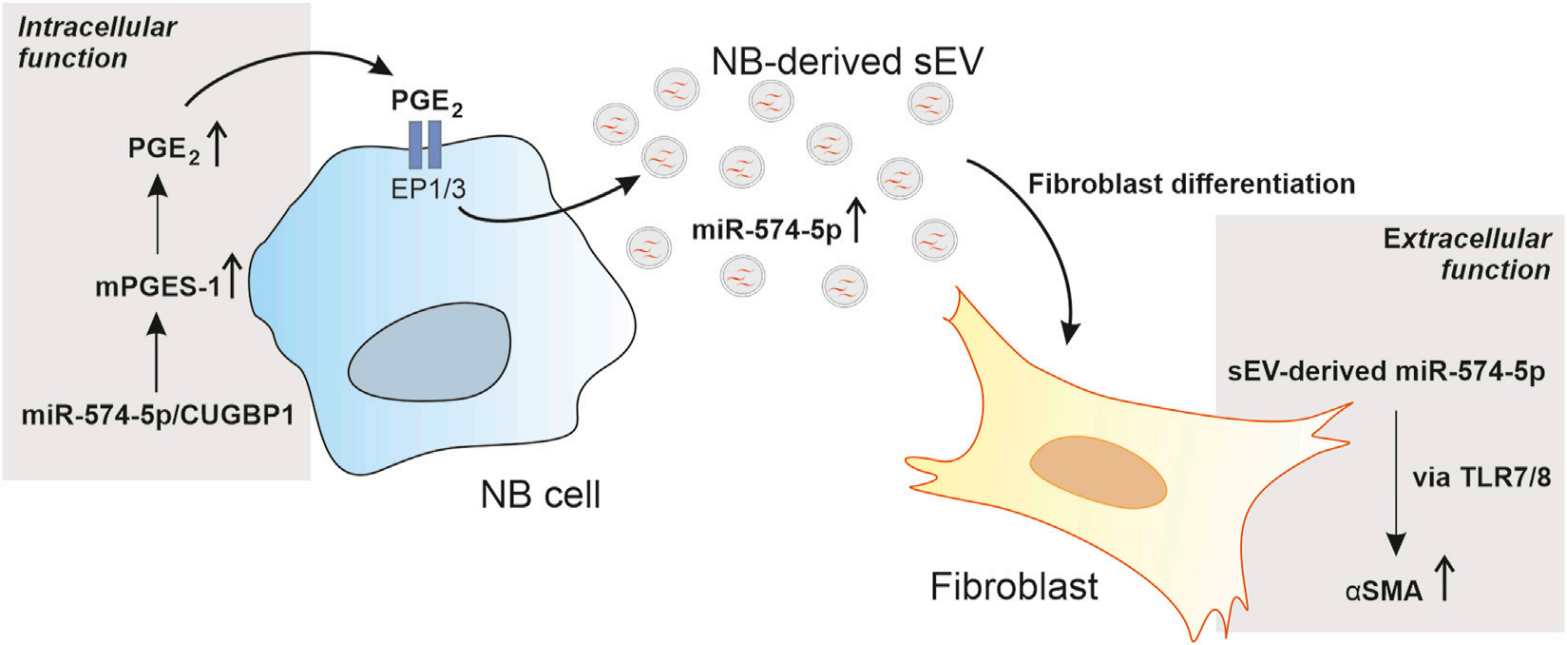
Schematic description of the multiple functions of miR-574-5p in the tumor microenvironment (TME) of neuroblastoma (NB). miR-574-5p activates prostaglandin E_2_ (PGE_2_) biosynthesis in tumor cells through interaction with CUGBP1. In response to PGE_2_ stimulation, this miR-574-5p is specifically sorted into small extracellular vesicles (sEV) and exerts a paracrine function in the TME. It modulates fibroblast differentiation via toll like receptor 7/8 (TLR7/8) signaling through induction of α-smooth muscle actin (α-SMA) expression.

Our data provide new insights into the multiple functions of miR-574-5p in the TME of neuroblastoma. We have shown that miR-574-5p activates PGE_2_ biosynthesis in tumor cells through interaction with CUGBP1. In response to PGE_2_ stimulation, this miRNA is specifically sorted into sEV and exerts a paracrine function in the TME of neuroblastoma by modulating fibroblast differentiation via TLR7/8 binding (Figure 6).

This is of particularly relevant, as CAFs have been described as major producers of mPGES-1-dependent PGE_2_ in neuroblastoma (Larsson et al., 2015; Kock et al., 2018). In our study, we confirmed that CAFs are predominantly positive for mPGES-1 but negative for miR-574-5p and CUGBP1. Notably, mPGES-1 inhibition in CAFs reduces tumor growth (Kock et al., 2018), suggesting a key role for intercellular communication (Kock et al., 2020). Unexpectedly, we found that mPGES-1 expression in differentiated neuroblastoma cells, presumably ganglion cells, correlates with miR-574-5p and CUGBP1 expression. We confirmed this observation in 3D tumor models and demonstrated a direct interaction between miR-574-5p/mPGES-1 and CUGBP1 in neuroblastoma spheroids. Neuroblastoma is the least differentiated and most malignant of the neuroblastic tumors, but it can also spontaneously transform into ganglioneuroblastoma or ganglioneuroma (Johnsen et al., 2019). Therefore, our observation that PGE_2_ biosynthesis in differentiating neuroblastoma cells is mediated by miR-574-5p interacting with CUGBP1 is intriguing. This raises the question of whether miR-574-5p regulates PGE_2_ biosynthesis, thereby positively influencing neuroblastoma differentiation.Overall, the tissue staining results are consistent with the performed spheroid and RIP experiments showing a direct interaction of CUGBP1 with miR-574-5p and mPGES-1. This supports the hypothesis that the interaction between miR-574-5p and CUGBP1 regulates mPGES-1-dependent PGE _2_ biosynthesis in neuroblastoma cells.

Next, we wanted to clarify to what extent the relative expression of CUGBP1 and miR-574-5p in the neuroblastoma is comparable to other PGE_2_-dependent tumors. Therefore, we included the A549 NSCLC cell line in our studies as a known model for miR-574-5p-mediated regulation of PGE_2_ synthesis (Saul et al., 2019b; Emmerich et al., 2020; Donzelli et al., 2021). In general, mPGES-1 levels were markedly lower in the SK-N-AS spheroid experiments compared to the A549 spheroid experiments. This correlates with an increased expression of CUGBP1 and a low level of miR-574-5p in the neuroblastoma spheroids. This may be a first indication that CUGBP1 regulates mPGES-1 expression in neuroblastoma and is not compensated by low miR-574-5p expression as in NSCLC. Despite these differences at the intracellular level, neuroblastoma and NSCLC share a common feature that contributes to cellular communication in the TME.

Upon stimulation with PGE_2_, both neuroblastoma and NSCLC cell lines specifically secreted miR-574-5p into sEV, which is mediated by the receptors EP1 and 3. Furthermore, we demonstrated that sEV-miR-574-5p functions as a TLR7/8 ligand in both tumor entities but induced different physiological processes in the tumor environment. sEV-miR-574-5p exerts an autocrine function in NSCLC by inhibiting PGE_2_ biosynthesis (Donzelli et al., 2021). In neuroblastoma, sEV-miR-574-5p has a paracrine function. By inducing the expression of α-SMA at the post-transcriptional level, it stimulated the differentiation of fibroblasts. Interestingly, lung cancer-specific sEV miR-574-5p cannot induce fibroblast differentiation. Conversely, neuroblastoma-specific sEV-miR-574-5p has no effect on PGE_2_ biosynthesis in neuroblastoma cells.

These results suggest that additional sEV-specific factors influence extracellular miR-574-5p function. Therefore, we analyzed the cell-specific properties of sEV populations. Our results show a unique composition of tetraspanin on the surface of sEV derived from SK-N-AS and SK-N-SH cells, which is not comparable to the properties of NSCLC sEV populations (Donzelli et al., 2021; Breitwieser et al., 2022). In our live cell experiments, we also observed cell-specific uptake of the sEV population by different cell types, suggesting that certain factors of the sEV influence the vesicle internalization process and thus the function of the sEV-derived miR-574-5p. Depending on the internalization mechanism, an sEV-derived miR can be released at different sites within the cell (McKelvey et al., 2015). This can have a significant impact on its function. By changing the uptake mechanism, miR-574-5p does not enter the endosome and cannot interact with TLR7/8. This is consistent with our previous results, where we obtained initial evidence that the sEV envelope is indeed important for miR-574-5p to function as a TLR7/8 ligand. (Hegewald et al., 2020; Donzelli et al., 2021). Further studies (Rana et al., 2012; Horibe et al., 2018) support our hypothesis that the tetraspanin composition on sEV is likely to influence target cell selection and the mechanism of sEV uptake. The expression of surface proteins and receptors on the cell membrane also has a strong influence on the uptake of sEV (Jadli et al., 2020). Therefore, we aimed to investigate whether differences in the composition of the tetraspanins CD9/CD63/CD81 could affect the function of sEV miR-574-5p. We therefore aimed to investigate whether differences in the composition of the tetraspanins CD9/CD63/CD81 could affect the function of sEV miR-574-5p. In general, the tetraspanins are a highly enriched family of membrane proteins that are important for a large number of cellular processes (Hemler, 2003). In fact, several recent studies suggest that tetraspanins are involved in forming and transporting sEV, membrane fusion, and target cell recognition (Hemler, 2005; Rana et al., 2012; van Niel et al., 2018; Larios et al., 2020). We used neutralizing antibodies to inhibit the function of specific tetraspanins CD9, CD63 and CD81 on the surface of sEV. In this way, we aimed to block the interactions between the surface proteins and the cells. Compared to tetraspanin knockdown, such an approach does not affect tetraspanin composition through the compensatory mechanism or sEV biogenesis (van Niel et al., 2011; Suárez et al., 2021). Blockade of CD63 and CD81 on SK-N-AS-specific sEVs resulted in a decrease in α-SMA levels in HFL1 cells, whereas blockade of CD63 on A549-specific sEVs resulted in a decrease in mPGES-1 levels. Next, we quantified uptake rates by different methods to determine whether the change in functionality was directly related to a change in sEV uptake. Our results show for the first time that the function of miR-574-5p is linked to the tetraspanin composition on the sEV surface. The vesicles not only transport miRs, but also appear to have an impact on their mode of action.

Overall, our study highlights the multiple ways in which miR-574-5p affects the microenvironment of PGE_2_-dependent tumors and the factors that influence miRNA function. Given the important role of sEV in NB progression, the role of sEV-miR-574-5p as a TLR7/8 ligand in neuroblastoma is of particular interest because of its impact on fibroblast differentiation. It is therefore tempting to speculate how miR-574-5p may also influence other cell types of the TME and whether this can be considered when developing new therapeutic strategies for children with neuroblastoma.

## Data Availability

The original contributions presented in the study are included in the article/Supplementary Material, further inquiries can be directed to the corresponding author.
