# Supplementary material for: The multiple functions of miR-574-5p in the neuroblastoma tumor microenvironment
Source: Front Pharmacol. 2023 Sep 4;14:1183720. doi: 10.3389/fphar.2023.1183720 (PMC10507178; doi:10.3389/fphar.2023.1183720)
Supplement: Supplementary file 1 [file DataSheet1.PDF]

Supplementary Material

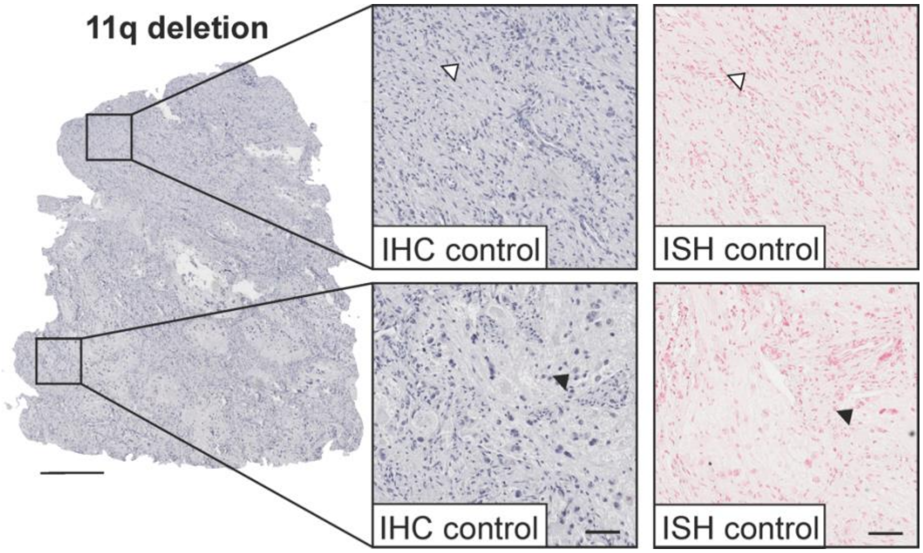

Figure S1: Negative controls immunostaining (IHC) and *In-situ* hybridization (ISH). Control IHC was performed using a rabbit IgG antibody and was counterstained with hematoxylin (blue). Control ISH was performed using a ScrC probe (blue) and sections were then counterstained with nuclear fast red (red). Sections from same neuroblastoma with 11q deletion as in Fig. 1 were used. Fibroblasts (white arrows) and differentiated tumor cells (black arrows) are highlighted. Scale bars: 0.5 mm, magnified images: 50 µm.

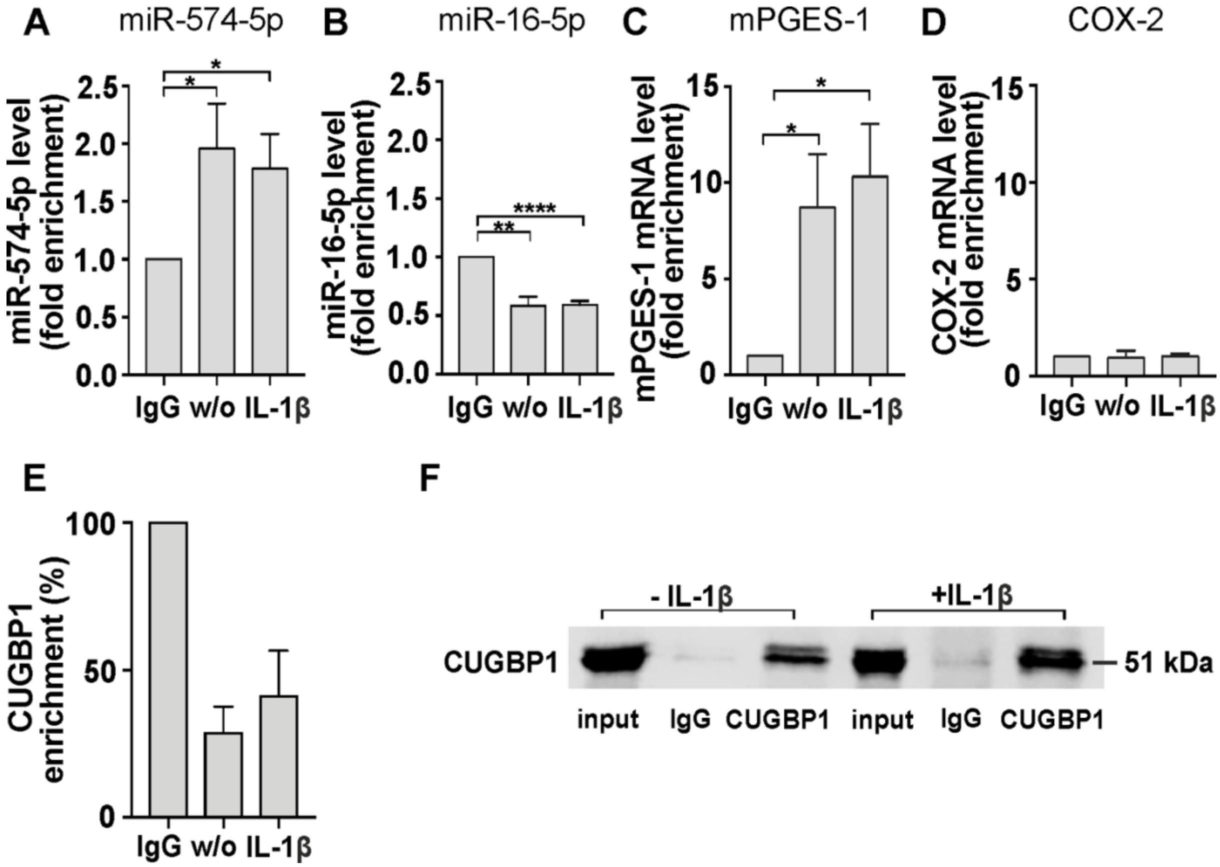

Figure S2: RNA-Immunoprecipitation of CUGBP1 from SK-N-AS cells reveals binding of miR-574-5p and mPGES-1 to CUGBP1. (A, B) MiR-574-5p enrichment was increased 1.96-fold to IgG control in the CUGBP1-immunoprecipitate of untreated cells and 1.78-fold in cells treated with 5 ng/mL interleukin (IL)-1β. MiR-16-5p was not enriched in the CUGBP1-immunoprecipitate. (C, D) MPGES-1 mRNA was enriched to 8.7-fold in the CUGBP1-immunoprecipitate of unstimulated cells and 10.31-fold in cells treated with IL-1β, whereas COX-2 was not enriched. (E, F) Validation of the CUGBP1-immunoprecipitation by Western blot using an α-CUGBP1 antibody. For untreated SK-N-AS cells 28.64% and for cells stimulated with IL-1β 41.01% of the total CUGBP1 were recovered in the immunoprecipitates. A representative blot of 4 independent experiments is shown. Data are presented as mean + SEM (N=4). Unpaired t-test to IgG control, $*p \leq 0.05$; $**p \leq 0.01$; $****p \leq 0.0001$.

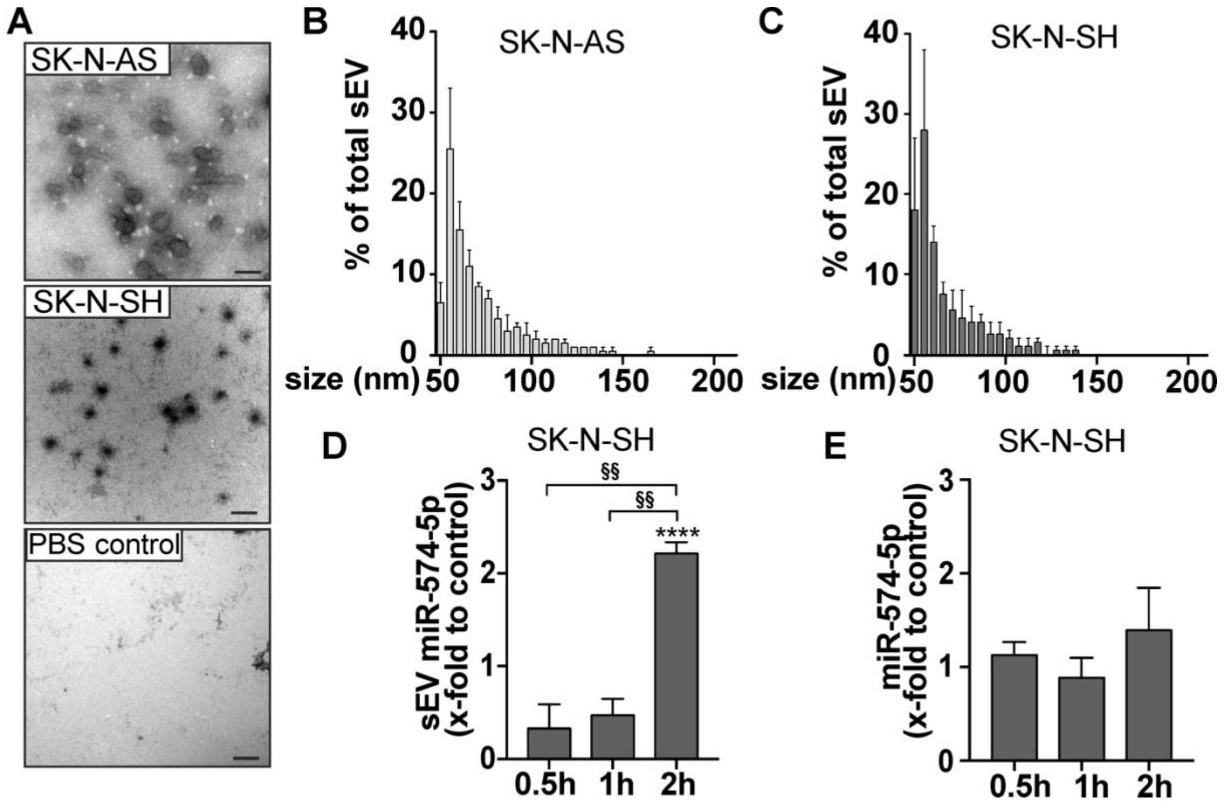

Figure S3: Characterization of neuroblastoma-derived sEV. (A) Proof of purification by transmission electron microscopy (TEM) of SK-N-AS and SK-N-SH sEV. SEV were isolated using differential ultracentrifugation. Scale bars: 200 nm. (B, C) size distribution was measured via interferometry vesicle sizing. Data are presented as mean + SEM of two independent experiments with each three technical replicates. (D, E) sEV-miR-574-5p secretion is measured in supernatants of SK-N-AS cells and SK-N-SH cells 30 min, 1 h and 2 h after 5 nM PGE_2 stimulation. MiR levels were analyzed by RT-qPCR, normalized to the spike-in control ath-miR-159a and folded to their corresponding control (N=4). Data are shown as mean +SEM, unpaired t-test to untreated control, ****$p \leq 0.0001$; unpaired t-test to other samples, §§ $p \leq 0.01$.

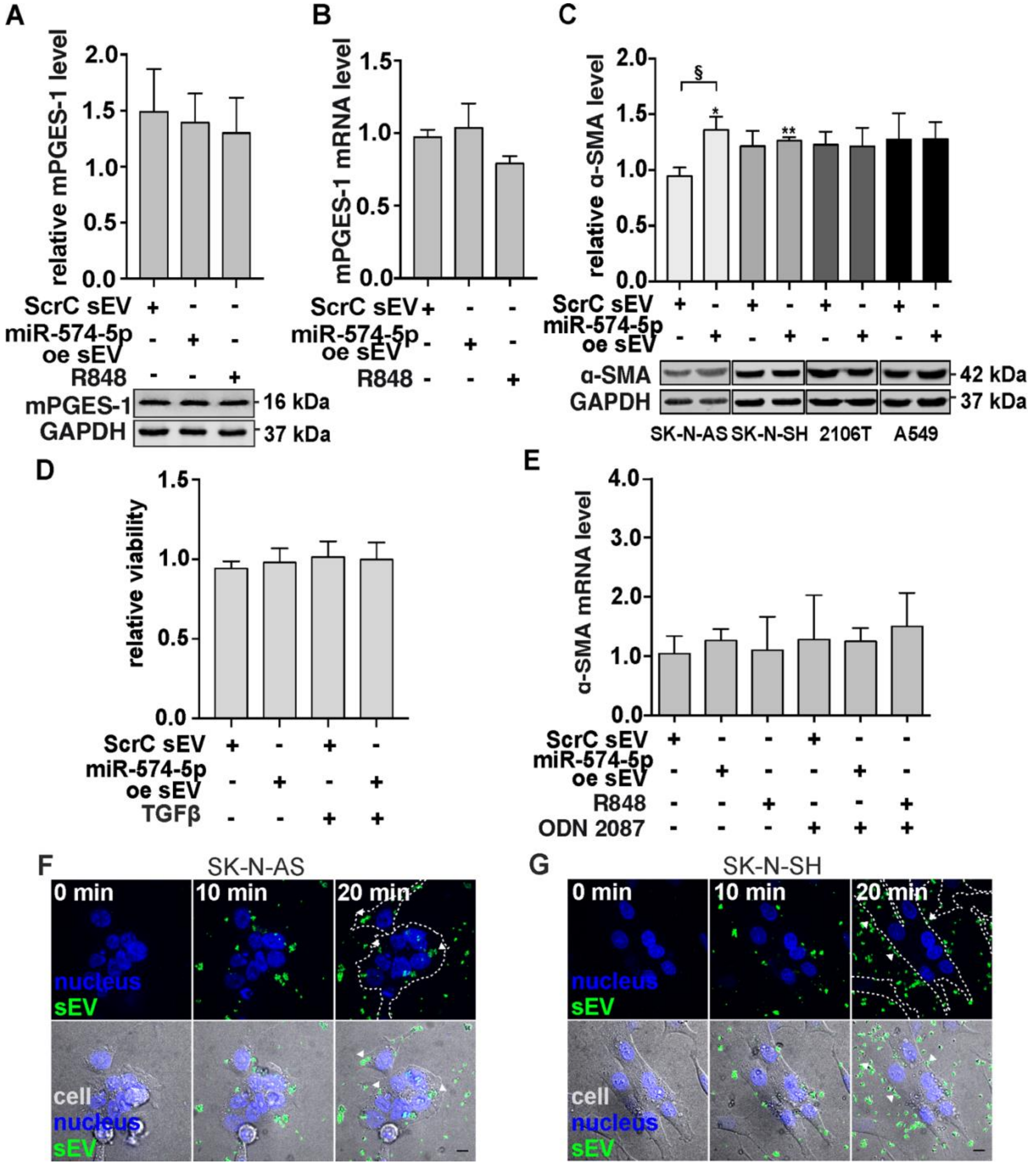

Figure S4: (A) Western blot analysis of SK-N-AS cells treated with miR-574-5p oe and ScrC sEV derived from SK-N-AS cells or 100 ng/mL TLR7/8 ligand R848. No significant effects on mPGES-1 protein level were detected. (B) RT-qPCR analysis of mPGES-1 mRNA of SK-N-AS stimulated with miR-574-5p oe or ScrC sEV derived from SK-N-AS cells or 100 ng/mL TRL7/8 ligand R848. No effects on the mPGES-1 mRNA level were detected. (C) Western blot analysis of human lung fibroblast (HFL1) cells treated with 2 µg/mL ScrC or miR-574-5p oe sEV derived from the cancer cell lines SK-N-AS, SK-N-SH, 2106T and A549 for 72 h. α-SMA levels were normalized to GAPDH and folded to untreated cell samples (SK-N-AS, A549, SK-N-SH: N=3, 2106T: N=4). Results are

shown as mean +SEM, unpaired t-test to control *p≤0.05; **p≤0.01; unpaired t-test to other samples. § p≤0.05. (D) Tetrazolium reduction assay of HFL1 cells after treatment with 2 µg/mL miR-574-5p oe or ScrC sEV and TGFβ. Effects on cell proliferation or metabolism were assessed with 3-(4,5-dimethylthiazol-2-yl)-2,5-diphenyltetrazoliumbromide (MTT, N=4). Results are shown as mean +SEM as x-folds to controls without sEV. (E) RT-qPCR analysis of α-SMA from HFL1 with miR-574-5p oe sEV. HFL1 cells were stimulated with 2 µg/mL of ScrC or miR-574-5p oe sEV derived from SK-N-AS cells or 100 ng/mL R848 and 200 nM ODN 2088 control (ODN 2087). Cells were harvested after 24 h and total RNA was extracted. Relative amounts of α-SMA mRNA were analyzed by RT-qPCR and GAPDH was used as endogenous control. The relative changes are shown as mean +SEM (N=6) as x-folds to control. (F, G) Live cell imaging of SK-N-AS and SK-N-SH cells and their miR-574-5p oe sEV. SK-N-AS took up their sEV after 20 min, while SK-N-SH sEV accumulated at the cell membrane. Representative images of three independent biologicals replicates with at least five technical replicates are shown. Scale bars: 10 µm

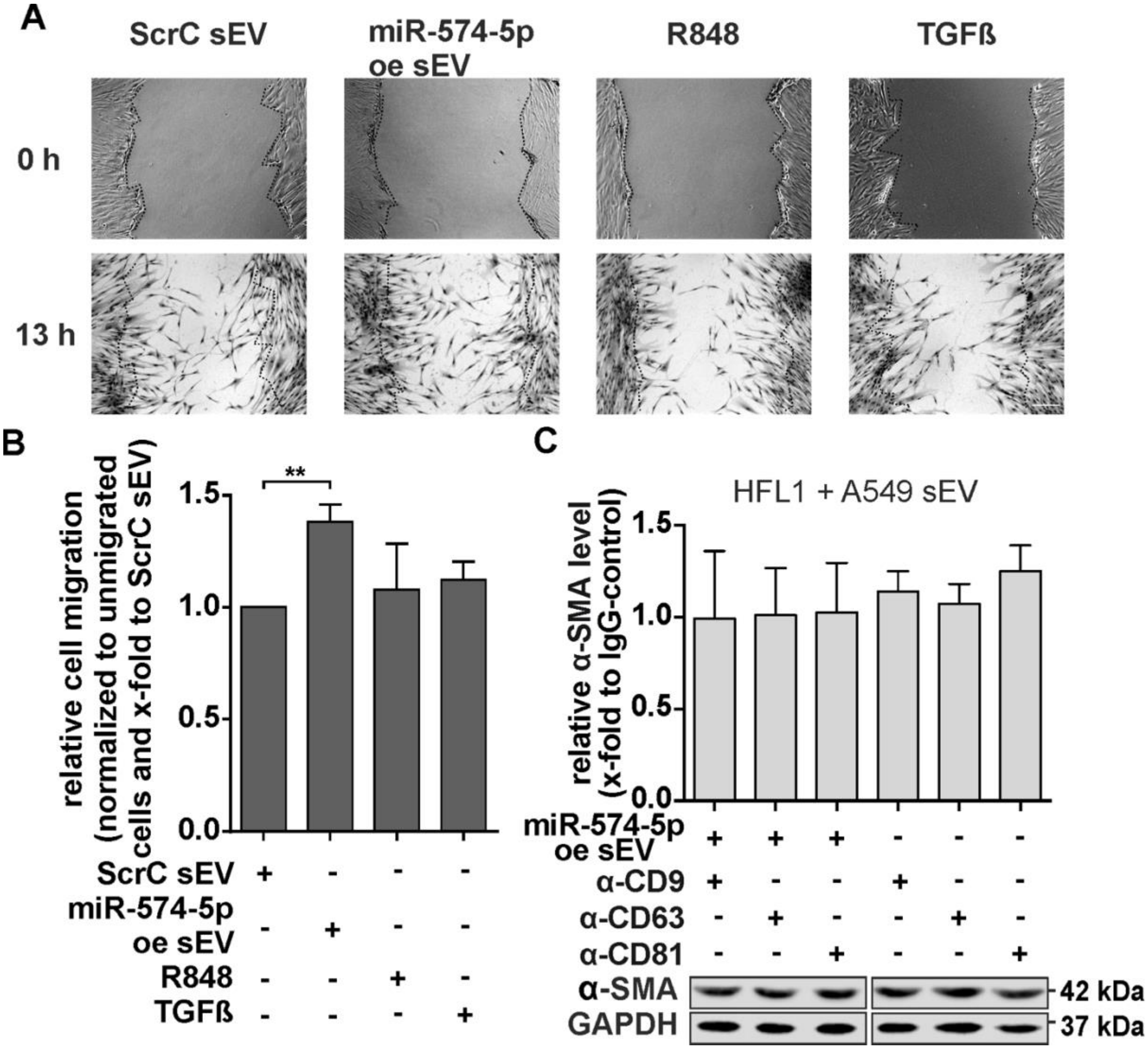

Figure S5: (A, B) Scratch-Assay of human lung fibroblast (HFL1) cells treated with miR-574-5p oe or ScrC sEV, 100 ng/mL R848 or 10 ng/mL transforming growth factor (TGF)-β. Cells were incubated with the different stimuli in starvation medium for 13 h after being scratched. Afterwards, nuclei were stained with hematoxylin and the number of cells that had migrated in between the scratch borders was counted with the ImageJ software and a cell counter plugin. Relative migration is shown as x-fold to ScrC sEV (N=4). Relative changes are shown as mean +SEM, unpaired t-test $^{**}p \leq 0.01$. Scale bar: 20 µm. (C) Western blot analysis of α-SMA protein levels in HFL1 cells treated with 2 µg/mL miR-574-5p oe sEV of A549 previously blocked with 50 ng α-CD9, α-CD63, α-CD81 or mouse IgG antibodies or only antibodies. Cells were treated with blocked sEV for 24 h. α-SMA levels were normalized to GAPDH and folded to IgG control samples (N=3).

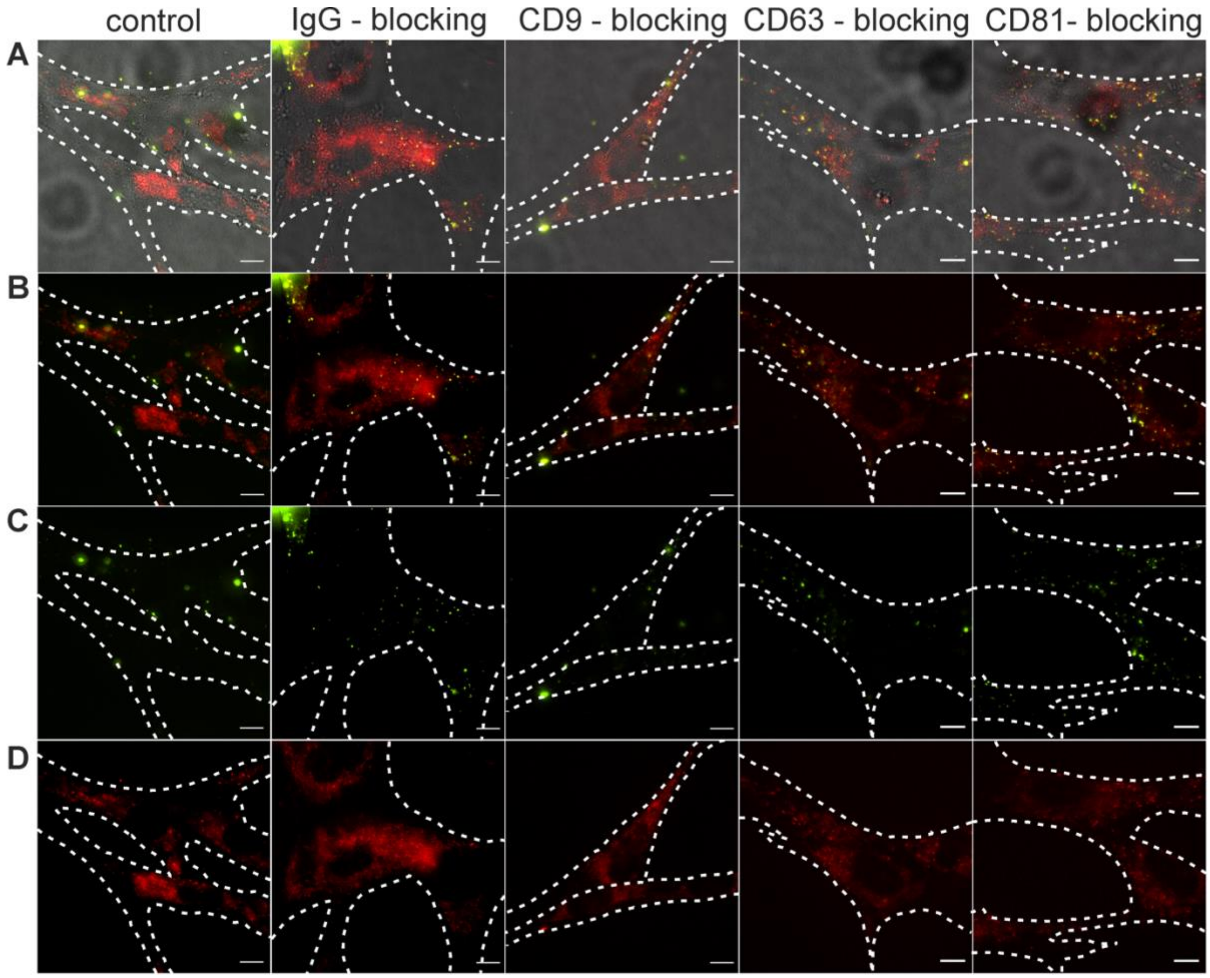

Figure S6: Live cell imaging of human lung fibroblast (HFL1) cells with neuroblastoma-derived miR-574-5p oe sEV. SK-N-AS-derived sEV were blocked with antibodies against CD9, CD63 and CD81. HFL1 cells were incubated for 1 h with DiO-labeled sEV together with 10µg/mL pHrodo™ Red Dextran and then monitored within 15 min. Representative images of (A) merge, (B) sEV and PHrodo™, (C) sEV, (D) PHrodo™ of three independent biological replicates with at least six technical replicates are shown. Scale bar: 10 µm

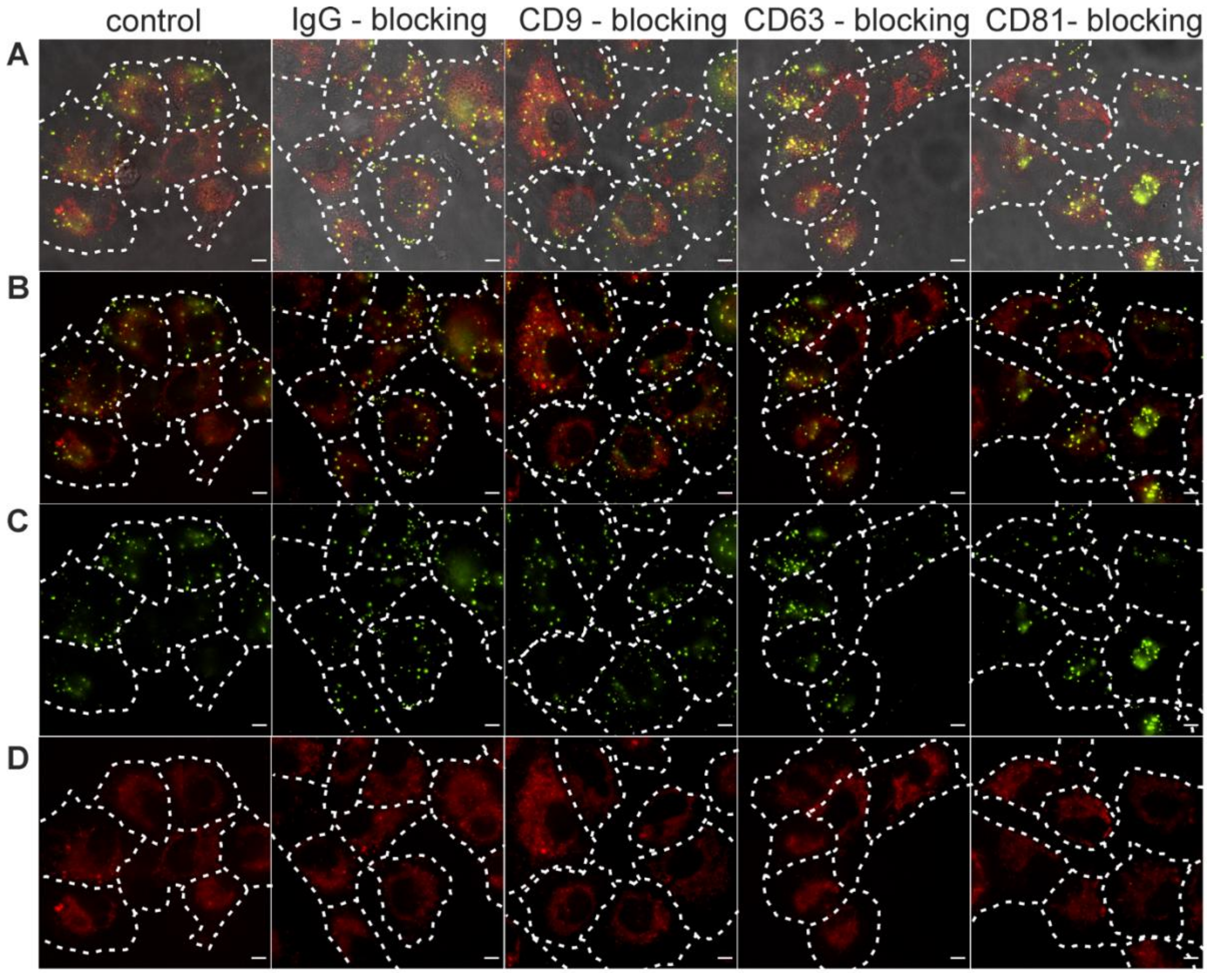

Figure S7: Live cell imaging of adenocarcinoma cells with their sEV. A549-derived sEV were blocked with antibodies against CD9, CD63 and CD81. A549 cells were incubated for 30 min with DiO-labeled sEV together with 10µg/mL pHrodo^{TM} Red Dextran and then monitored within 15 min. Representative images of (A) merge, (B) sEV and PHrodo^{TM}, (C) sEV, (D) PHrodo^{TM} of three independent biological replicates with at least six technical replicates are shown. Scale bar: 10 µm

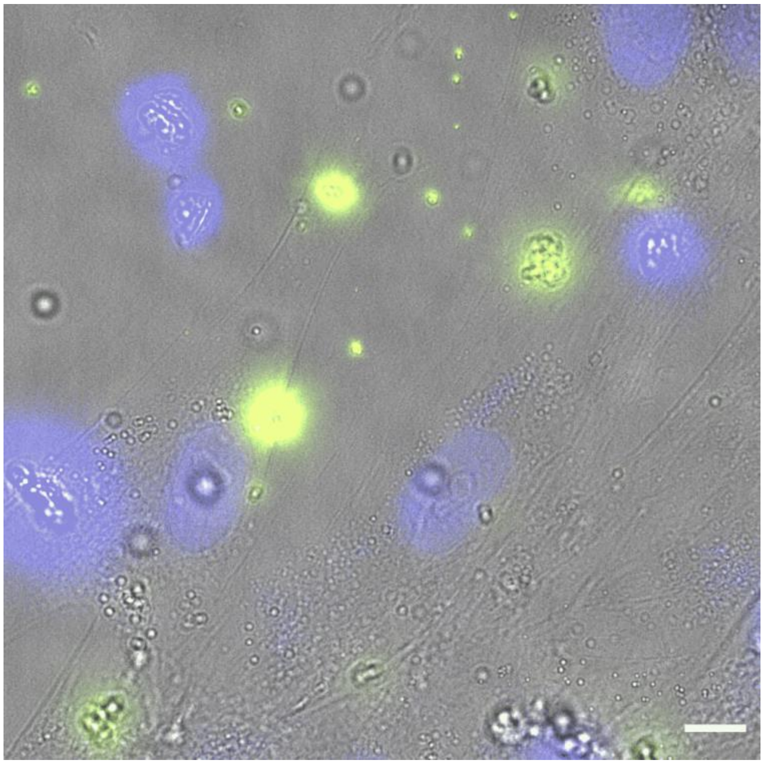

Video S1: Uptake of neuroblastoma sEV by human lung fibroblast (HFL1) cells. SK-N-AS-derived sEV were labeled with DiO and nuclei with Hoechst. HFL1 cells were incubated with labeled sEV for 50 minutes and uptake was monitored.
